# Historical evolution, research hotspots and emerging trends of pediatric hand, foot, and mouth disease: a bibliometric worldview since the 21st century

**DOI:** 10.3389/fmed.2025.1722750

**Published:** 2025-12-05

**Authors:** Lei Liang, Yujing Zhang, Xinxin Zhang, Xuran Guo, Yongbin Yan

**Affiliations:** 1 School of Pediatric Medicine, Henan University of Traditional Chinese Medicine, Zhengzhou, Henan, China; 2 First Affiliated Hospital of Henan University of Traditional Chinese Medicine, Zhengzhou, Henan, China

**Keywords:** HFMD, children, enteroviruses, coxsackieviruses, VOSviewer, CiteSpace, visual analysis

## Abstract

**Background:**

Hand, foot, and mouth disease (HFMD) poses a significant challenge to global public health. Primarily caused by enterovirus and coxsackievirus infections, the disease has a particularly pronounced impact in the Asia-Pacific region. However, systematic analysis and discussion regarding the developmental trajectory, core research entities, current status, key research directions, and future prospects of pediatric HFMD research remain lacking.

**Methods:**

This study collected and analyzed papers and reviews on pediatric HFMD published between January 1, 2000, and February 1, 2025, from the Web of Science Core Collection and PubMed. Key research indicators were analyzed through bibliometric visualization, using tools including Excel, CiteSpace, VOSviewer, and BibliomeTools (an R-based tool in R-Studio).

**Results:**

Since the start of the 21 st century, academic publications in pediatric HFMD have steadily increased, with a cumulative total of 2,034 papers published by February 1, 2025. Global research distribution exhibits uneven patterns, with China emerging as core contributors. Specifically, Lin, Tzou-Yien from China, has published the largest number of papers, while Chang, Luan-Yin is the co-cited author with the highest citation rate. Solomon T et al.’s “Virology,” Epidemiology, Pathogenesis, and Control of Enterovirus 71” being the most cited study in the field. Research on pediatric HFMD is closely integrated with disciplines such as virology and epidemiology, forming core research themes around “HFMD,” “enterovirus 71,” and “enteroviruses.” Recent research has focused on the pathogenesis, epidemiology, novel therapeutic discoveries and vaccine development for pediatric HFMD. Looking ahead, it is essential to delve deeper into the molecular mechanisms underlying the interaction between the human HFMD virus and its host, and to develop multivalent vaccines targeting multiple serotypes.

**Conclusion:**

This study employs bibliometric methods to visualize research in the field of pediatric hand, foot, and mouth disease, revealing trends and frontiers in this area. It will provide valuable reference for scholars seeking key research questions and potential collaborators.

## 1 Introduction

Hand, foot and mouth disease is a common infectious disease in children caused by enterovirus infection. Children under 5 years of age, especially those under 3 years, have the highest incidence. Since its first report in New Zealand in 1957, HFMD has spread widely around the world (1). In most cases, the symptoms of HFMD are mild and manifest as fever and vesicular rashes on the hands, feet, and mouth, usually lasting less than a week (2). However, many patients exhibit critical symptoms, often severe neurological lesions and cardiopulmonary complications, and may experience long-term neurological sequelae after recovery (3). Therefore, HFMD is regarded as a serious public health problem globally. The disease is concentrated mainly in regions such as Asia, Africa, and South America. China is a major high-incidence area, which is related to the warm and humid climate in these regions, which is conducive to the survival and spread of enteroviruses (4). Moreover, in some developing countries, poor sanitation conditions, insufficient public health facilities, and low levels of health education also increase the risk of virus transmission. Recently, a tomato flu, also caused by an enterovirus, was discovered in India. Owing to its symptoms being similar to those of HFMD, people have once again focused on the epidemic of HFMD (5). In terms of virus types, various types of enteroviruses cause HFMD. Coxsackievirus A16 (CVA16) and enterovirus 71 (EV71) are the most common, and they are the main virus types causing severe cases and deaths (6). However, in recent years, the number of cases caused by Coxsackievirus A6 (CVA6) and Coxsackievirus A10 (CVA10) has shown an increasing trend in some regions (7). To date, many scholars have researched the pathogenesis (8), epidemiology (9), and related treatment and prevention measures (8, 10) of HFMD. Vaccines against EV71 have also achieved remarkable results (11). However, with the publication of many research findings, there is a lack of systematic analysis of academic achievements and research status in this field from a global perspective. Moreover, systematic reviews and meta-analyses cannot predict the future development trend of a field, making it difficult for researchers to capture the current research hotspots, key and difficult points, and future trends.

Bibliometrics emerged in the 1960s. As an interdisciplinary science, it uses mathematical and statistical methods to conduct quantitative analysis of various knowledge carriers and is an important part of the information science system. Compared with systematic literature reviews, bibliometrics can provide more objective and reliable analysis results, effectively reducing potential biases caused by subjective intentions; thus, bibliometrics has been widely applied in academic research (12–14). In research related to pediatric HFMD, many previous clinical and basic studies have made significant contributions to the development of this field. However, bibliometric analysis in this field is relatively rare. Therefore, this study systematically collected and analyzed the academic literature related to pediatric HFMD in recent years in detail, aiming to comprehensively reveal the research trends and development trends in this field. This study used methods such as keyword co-occurrence analysis, citation analysis, and author–institution cooperation network analysis to explore the main themes, core researchers, key research institutions, and geographical distribution of research focused on the study of pediatric HFMD in detail. In addition, this study also evaluated the knowledge base and research frontiers of research in this field to provide valuable reference and guidance for future related research.

## 2 Methods

### 2.1 Data source and search strategy

**FIGURE 1:**
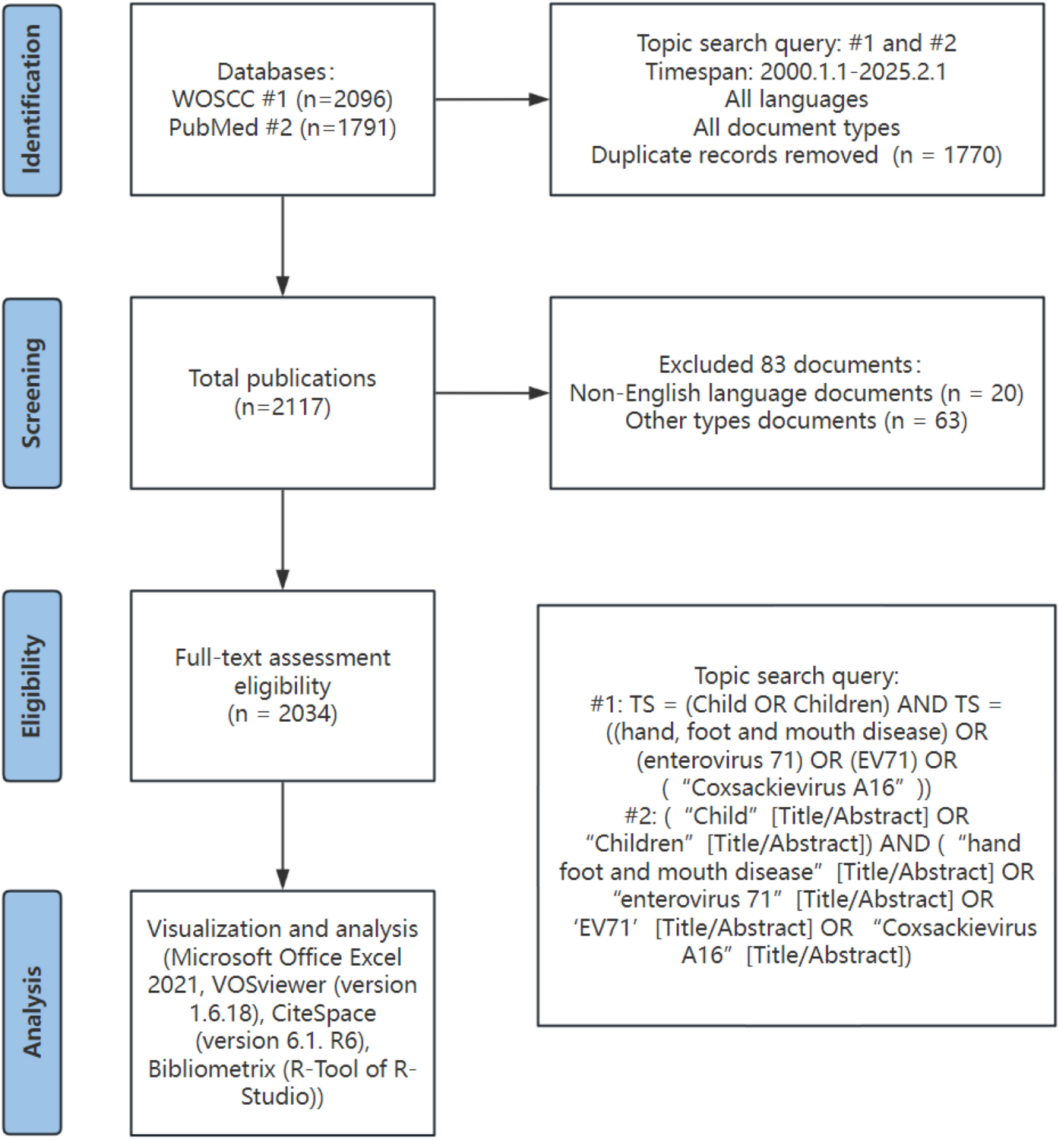
Flowchart of the literature screening process for pediatric hand, foot, and mouth disease included in this study.

In this study, all literature data were sourced from the Web of Science Core Citation and PubMed (15–17). These two databases encompass over 12,000 influential academic journals, whose validity has been verified by numerous researchers conducting bibliometric analyses. The time span for the bibliometric analysis in this paper is from January 1, 2000, to February 1, 2025. The search formula employed by Web of Science Citation Citation (WOSCC) is as follows: TS = (Child OR Children) AND TS = ((hand, foot and mouth disease) OR (enterovirus 71) OR (EV71) OR (“Coxsackievirus A16”)); The PubMed search formula is: (“Child”[Title/Abstract] OR “Children”[Title/Abstract]) AND (“hand foot and mouth disease”[Title/Abstract] OR “enterovirus 71”[Title/Abstract] OR ‘EV71’[Title/Abstract] OR “Coxsackievirus A16”[Title/Abstract]). Among the above results, we retrieved 2,096 articles from WOSCC and 1,791 articles from PubMed. After removing 1,770 duplicate articles, excluding 20 non-English articles, and filtering out 63 non-article and non-review publications, we ultimately obtained 2,034 articles and reviews for subsequent bibliometric analysis (Figure 1).

### 2.2 Data analysis

After confirming the accuracy of the data, the screened and optimized original dataset was exported in the format of a.txt file. This dataset contains important information such as titles, authors, keywords, institutions, countries/regions, citations, journals, and publication dates. This study utilized tools such as Microsoft Office Excel 2021, VOSviewer (version 1.6.18), CiteSpace (version 6.1. R6), and the R package “Bibliometrix” was used to conduct the data analysis and visualization.

**FIGURE 2:**
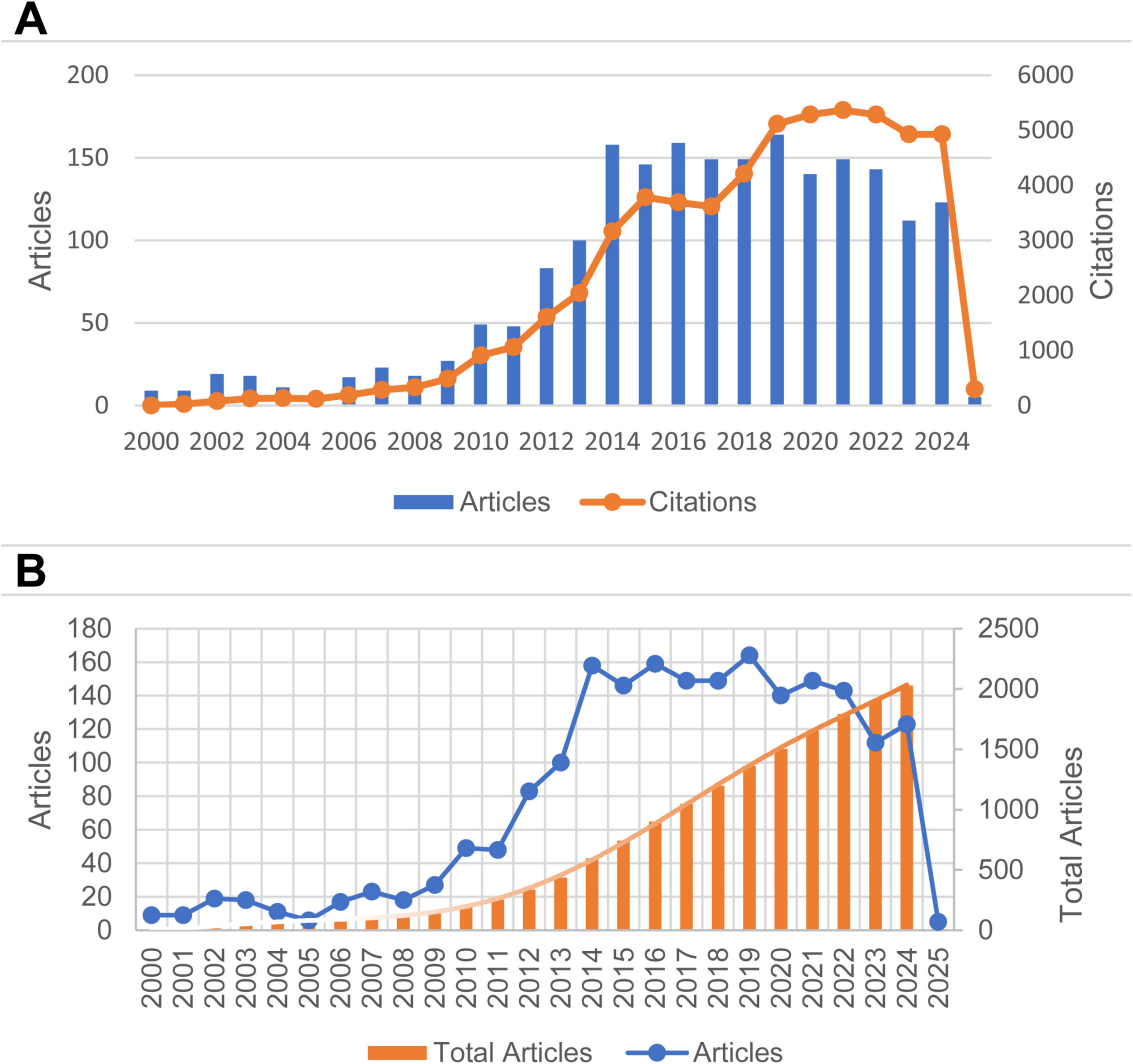
**(A)** The annual publication quantity and citation frequency of pediatric HFMD research from 2000 to 2025. **(B)** The annual publication quantity, cumulative publication quantity, and polynomial fitting curves for pediatric HFMD research from 2000 to 2025.

As the core spreadsheet component within the Office 2021 suite, Microsoft Office Excel 2021 adapts to diverse data analysis and processing scenarios across multiple fields. This study imported bibliometric data and foundational literature information from the target field into Microsoft Office Excel 2021. After data organization and classification, analytical tables required for the paper were generated. Furthermore, based on the aforementioned publication volume and citation data, Figure 2 was plotted using this software. Simultaneously, a polynomial fitting method was employed to conduct trend analysis on the annual cumulative publication volume data.

CiteSpace (18, 19) was developed by Chaomei Chen et al. Its principle is to create a network map of a specific field, thereby obtaining key information about the research in that field, such as potential trends, research frontiers, and research directions. In this study, with the help of CiteSpace software, co-occurrence and cluster analyses were carried out for information such as authors, research institutions, and countries. The Citespace analysis parameters used in this study are as follows: Link Retaining Factor (LRF) = 3.0; Maximum Links Per Node (L/N) = 10; Look Back Years (LBY) = 5; Percentage of Nodes to Label = 1.0%; Threshold (k) = 25; Clustering quality indices: Modularity Q > 0.5, Weighted Mean Silhouette S > 0.7, indicating good clustering quality.

VOSviewer (20, 21) is a bibliometric analysis software developed by Nees Jan van Eck et al. in that is mainly applied in the data extraction and processing stages. In this study, VOSviewer was used to analyze the distribution of countries/regions, institutions, author collaboration and distribution, as well as the distribution and collaborative relationships of keywords. The VOSviewer analysis parameters used in this study are as follows: Keyword co-occurrence analysis: Minimum number of occurrences = 6; Author co-authorship analysis: Minimum number of publications = 9; Co-cited author analysis: Minimum number of co-citations = 75; Co-cited journal analysis: Minimum number of co-citations = 100; Country collaboration analysis: Minimum number of publications = 5; Journal analysis: Minimum number of publications = 5.

Bibliometrix (22–24) is an R package developed by Aria, Cuccurullo et al. in and is mainly used for comprehensive bibliometric and scientometric analyses. In this study, we used Bibliometrix to analyze the evolutionary trends of keywords in the literature. Through the comprehensive use of the abovementioned tools, we achieved multidimensional analysis and visual display of the literature information, which not only adhered to academic norms but also ensured the scientific validity and effectiveness of the research.

## 3 Results

### 3.1 Publication and citation analysis

Changes in the volume of scientific literature at specific points in time can reveal the accumulation of knowledge within a particular research field, providing crucial parameters for quantitatively assessing its development. Figure 2A shows the trends in the number of publications and citation counts in the field of pediatric HFMD research from 2000 to 2025. In terms of the number of publications, before 2009, the number of publications in this field remained relatively low. From 2009 to 2014, the number of annual publications rapidly increased, reaching a maximum of 164 in 2019. The citation counts experienced rapid upward phases between 2009–2015 and 2017–2019, with a peak of 5,365 citations in 2021. Notably, the data collection for this study was completed in early February 2025, so the number of publications and citation counts for 2025 cannot be reflected, and the data for this year lack reference value to a certain extent.

In this work, polynomial fitting was also carried out on the annual cumulative number of publications, as shown in Figure 2B. The fitting formula is y = 0.0002x^6^ −0.0158x^5^ + 0.4367x^4^ −5.125x^3^ + 27.041x^2^ −45.13x + 33.066, and the goodness of fit is R^2^ = 0.9998. This fitted curve shows a favorable upward trend and has a high goodness of fit. This result fully demonstrates the broad development prospects of this research field.

### 3.2 Distribution of countries/regions

This study analyzes 78 countries and regions participating in pediatric HFMD research to identify key contributors and the distribution of academic centers in this field. Table 1 lists the top 10 countries and regions in terms of publication volume, citation count, and Total Link Strength in pediatric HFMD research. China holds an absolute dominant position in this field, leading significantly with 1,457 publications (71.63%) and 37,765 citations. This dominance is closely linked to the historical prevalence of HFMD in China. Beyond China, significant contributions from countries such as the USA (221 papers, 10.87%, 8,496 citations) and the UK (106 papers, 5.21%, 5,541 citations) have been crucial in advancing this research domain.

**TABLE 1:** Ranking of the top ten major countries/regions of pediatric HFMD research from 2000 to 2025.

Rank	Countries	Documents	Countries	Citations	Countries	Total link strength
1	China	1,457	China	37,765	China	329
2	USA	221	USA	8,496	USA	212
3	UK	106	UK	5,541	UK	159
4	Singapore	90	Malaysia	4,620	Vietnam	92
5	Australia	74	Singapore	3,907	Australia	79
6	Malaysia	73	Australia	3,827	Singapore	66
7	Japan	58	Japan	2,365	France	61
8	France	53	Vietnam	2,071	Malaysia	58
9	Vietnam	51	France	1,414	Japan	49
10	India	43	Finland	920	Netherlands	35

**FIGURE 3:**
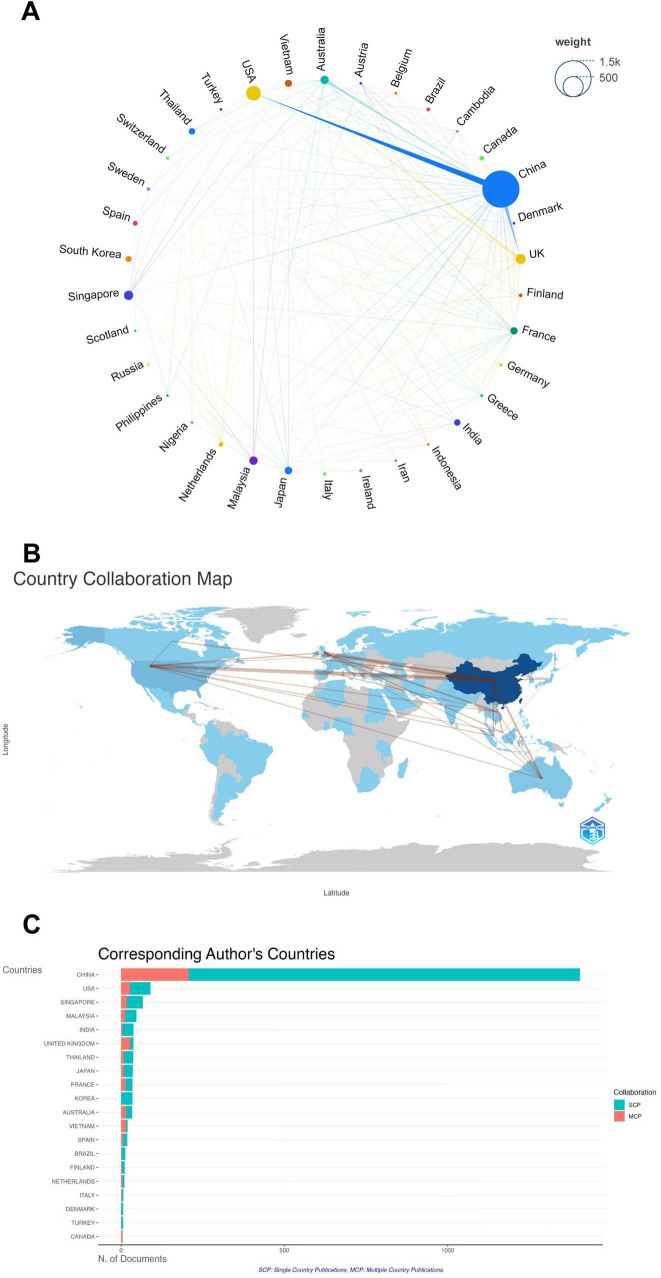
**(A)** The contributions of different countries to research on pediatric HFMD are presented visually, with each country represented by a colored dot. The connecting lines between the dots indicate academic exchanges, and the thickness of the lines is directly related to the frequency of academic interactions between the countries. **(B)** Geographic distribution of major literature-producing countries in the field of pediatric HFMD research. This study highlights the academic collaboration between these countries and provides a clear view of global academic relationships. The brown lines in the figure indicate academic collaborations, and the thickness of the lines indicates the frequency of exchanges between the countries involved. **(C)** The proportion of single-country publications to multiple-country publications among the top twenty countries in terms of publication output indirectly reflects the academic collaboration tendencies of these countries.

Figure 3A shows the academic achievements of various countries in the research field of pediatric HFMD and the academic cooperation network among countries. An analysis of the connecting lines clearly reveals that there are relatively close academic connections between China and the United States, between China and Australia, and between China and the United Kingdom. This strongly reflects that the activity and participation levels of these countries and regions in the research field of pediatric HFMD are relatively high. Figure 3B vividly and three-dimensionally presents the geographical distribution pattern of the main literature-producing countries in this field. China, the United States, the United Kingdom, and Australia clearly play crucial roles as academic hubs in this research field. They have established close academic connections with many other countries and play an important role in promoting academic development and exchanges in this field. In addition, Figure 3C shows in detail the number of papers produced by each country in the research field of pediatric HFMD and uses different colors to distinguish between studies published through transnational cooperation and those coauthored by domestic authors. In countries with a large number of published papers, such as China, the United States, and Singapore, although they have extensive cooperation with other countries, most of the published literature still comes from cooperation among domestic authors. In contrast, British authors tend to establish transnational cooperation in academic exchanges.

### 3.3 Distribution of institutions

To assess the academic influence and contributions of relevant institutions and reveal their collaborative networks and patterns, we compiled and analyzed data from 2,234 institutions engaged in research within this field. Table 2 lists the top 10 institutions by number of published papers and citation counts. Chinese Academy of Sciencess leads in publication volume with 120 papers, followed closely by the Chinese Center for Disease Control and Prevention with 118 papers. The latter also tops the citation list with 5,063 citations. Beyond these two institutions, Chang Gung University (76 papers) and Fudan University (74 papers) also rank highly in paper output. Outside of China, overseas institutions such as the National University of Singapore (67 papers, 2,598 citations) and the University of Oxford (55 papers, 2,578 citations) also made significant contributions in this field.

**TABLE 2:** Ranking of the top ten major institutions of pediatric HFMD research from 2000 to 2025.

Rank	Institution	Publications	Original country	Institution	Citations	Original country
1	Chinese academy of sciences	120	China	Chinese center for disease control and prevention	5,063	China
2	Chinese center for disease control and prevention	118	China	Chang Gung University	3,963	China
3	Chang Gung University	76	China	Chinese Academy of Sciences	3,801	China
4	Fudan University	74	China	National Cheng Kung University	3,755	China
5	National Cheng Kung University	67	China	Universiti Malaysia Sarawak	2,744	Malaysia
6	National University of Singapore	67	Singapore	National University of Singapore	2,598	Singapore
7	Chinese Academy of Medical Sciences	64	China	University of Oxford	2,578	UK
8	National Health Research Institutes	57	China	University of Liverpool	2,250	UK
9	University of Oxford	55	UK	National Institutes for Food and Drug Control	2,245	China
10	National Taiwan University	49	China	National Health Research Institutes	2,227	China

**FIGURE 4:**
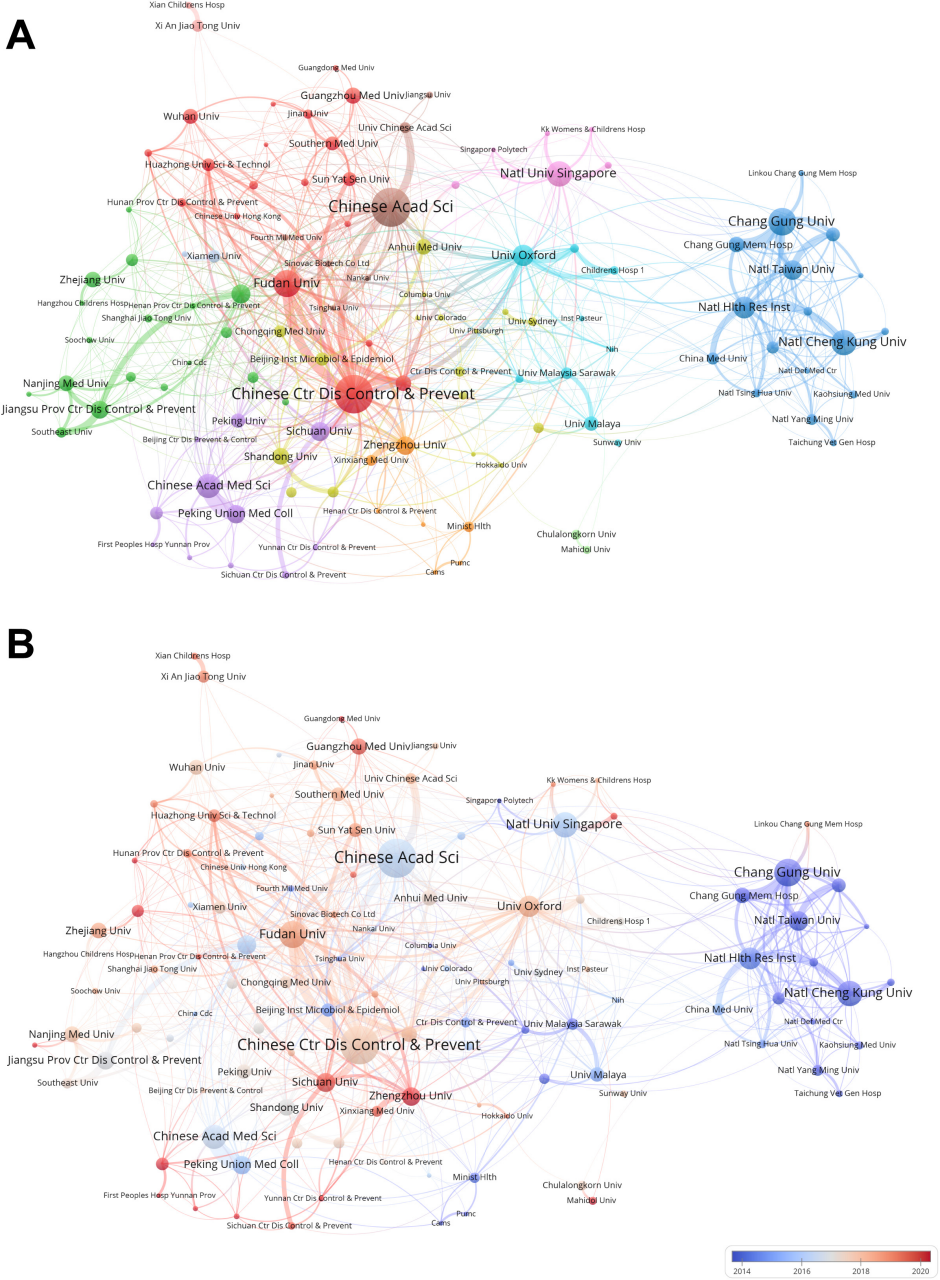
**(A)** Institutional cluster analysis. Node colors indicate different clusters, node diameters indicate the number of articles published by the institutions, and line thicknesses indicate the degree of institutional collaboration. **(B)** Graph showing the recent contributions of institutions in the field of pediatric HFMD research compared with their overall output. The color scale reflects the level of institutional activity over the past decade, with red indicating increased impact and blue indicating decreased activity in the area, highlighting institutions that have had a significant impact or decreased involvement in the research area.

Furthermore, we used VOSviewer to generate Figure 4 to visually display the potential connections between research institutions. In Figure 4A, the cooperative relationships between these institutions show significant geographical characteristics. Institutions in the Taiwan Province of China are mainly concentrated in the blue cluster on the right side of the figure, including National Cheng Kung University and National Health Research Institutes. The clusters on the left side of the figure are mainly composed of institutions in mainland China. For example, the Chinese Academy of Sciences is in the brown cluster, the Chinese Center for Disease Control and Prevention and Fudan University are in the red cluster, Zhengzhou University is in the orange cluster, Shandong University is in the yellow cluster, and so on. In addition, the pink cluster includes institutions in Singapore, such as KK Women’s and Children’s Hospital, Singapore Polytechnic, and the National University of Singapore. The cyan cluster encompasses institutions from countries such as Malaysia and the UK, such as the University of Malaysia Sarawak, the University of Malaya, and the University of Oxford. Notably, the clusters of institutions in mainland China not only have close internal connections but also have constructed a complex cooperation network among different clusters. In contrast, institutions in the Taiwan Province of China have fewer external connections. In terms of the chronological order of cooperation, Figure 4B shows that institutions in the Taiwan Province of China established academic connections earlier, whereas cooperation among institutions in mainland China started relatively later. Zhengzhou University and Sichuan University have only become active in cooperative research in this field in recent years.

### 3.4 Distribution of authors and co-cited authors

Analyzing authors and co-cited authors helps us identify core contributors within a field, uncover academic connections and collaboration networks among authors, and reveal the knowledge base and academic lineage of the field. Table 3 lists the top 10 authors in terms of the number of published papers and co-citation counts. Among them, all the top 10 authors by the number of published papers are from China. Lin, Tzou-Yien from Kaohsiung Chang Gung Memorial Hospital published 40 papers; Liang, Zhenglun and Mao, Qunying from the National Institutes for Food and Drug Control published 37 and 36 papers, respectively. In terms of co-citation counts, Chang Ly from National Taiwan University has received the most attention, with 785 co-citations; followed by Wang Sm from National Cheng Kung University, with 700 co-citations; and Zhang Y from the Chinese Academy of Medical Sciences, with 666 co-citations. The above phenomena once again confirm the significant influence of China in the research field of pediatric HFMD. Supplementary Figure 1A shows the trends in the number of publications and total citation counts of key authors over time in the research field of pediatric HFMD. Among them, Lin, Tzou-Yie, who has the highest number of publications in this field, is also the author with the longest time span of creation.

**TABLE 3:** Ranking of the top 10 major authors of pediatric HFMD research from 2000 to 2025.

Rank	Author	Documents	Total link strength	Countries/ regions	Institution	Author	Co citations	Total link strength	Countries/ regions	Institution
1	Lin, Tzou-Yien	40	115	China	Kaohsiung Chang Gung Memorial Hospital	Chang, Ly	785	1,1913	China	National Taiwan University
2	Liang, Zhenglun	37	155	China	National Institutes for Food and Drug Control	Wang, Sm	700	10,049	China	National Cheng Kung University
3	Mao, Qunying	36	157	China	National Institutes for Food and Drug Control	Zhang, Y	666	10,508	China	Chinese Academy of Medical Sciences
4	Chang, Luan-Yin	35	80	China	National Taiwan University	Zhu, Fc	541	8,470	China	Southeast University-China
5	Zhu, Fengcai	31	100	China	Southeast University-China	Solomon, T	514	7,171	UK	University of Liverpool
6	Shih, Shin-Ru	30	43	China	Chang Gung University of Science and Technology	Ho, Mt	501	7,194	China	National Health Research Institutes
7	Liu, Ching-Chuan	27	60	China	National Cheng Kung University	Ooi, Mh	493	7,193	Malaysia	University of Malaysia Sarawak
8	Duan, Guangcai	26	105	China	Zhengzhou University	Xing, Wj	416	5,082	China	Taiyuan University of Technology
9	Huang, Zhong	26	80	China	Fudan University	Lin, Ty	373	6,146	China	Kaohsiung Chang Gung Memorial Hospital
10	Jin, Yuefei	26	105	China	Zhengzhou University	Oberste, Ms	347	3,789	USA	Centers for Disease Control and Prevention

Lotka’s law, one of the earliest well-known laws in the field of bibliometrics, plays an important role in research (25). As shown in Supplementary Figure 1B, a comparison reveals that the proportion of authors with a small number of published papers is greater than the expected value. This indicates that in the research field of pediatric HFMD, the number of authors who have long been deeply engaged in and committed to research in this field is relatively limited.

**FIGURE 5:**
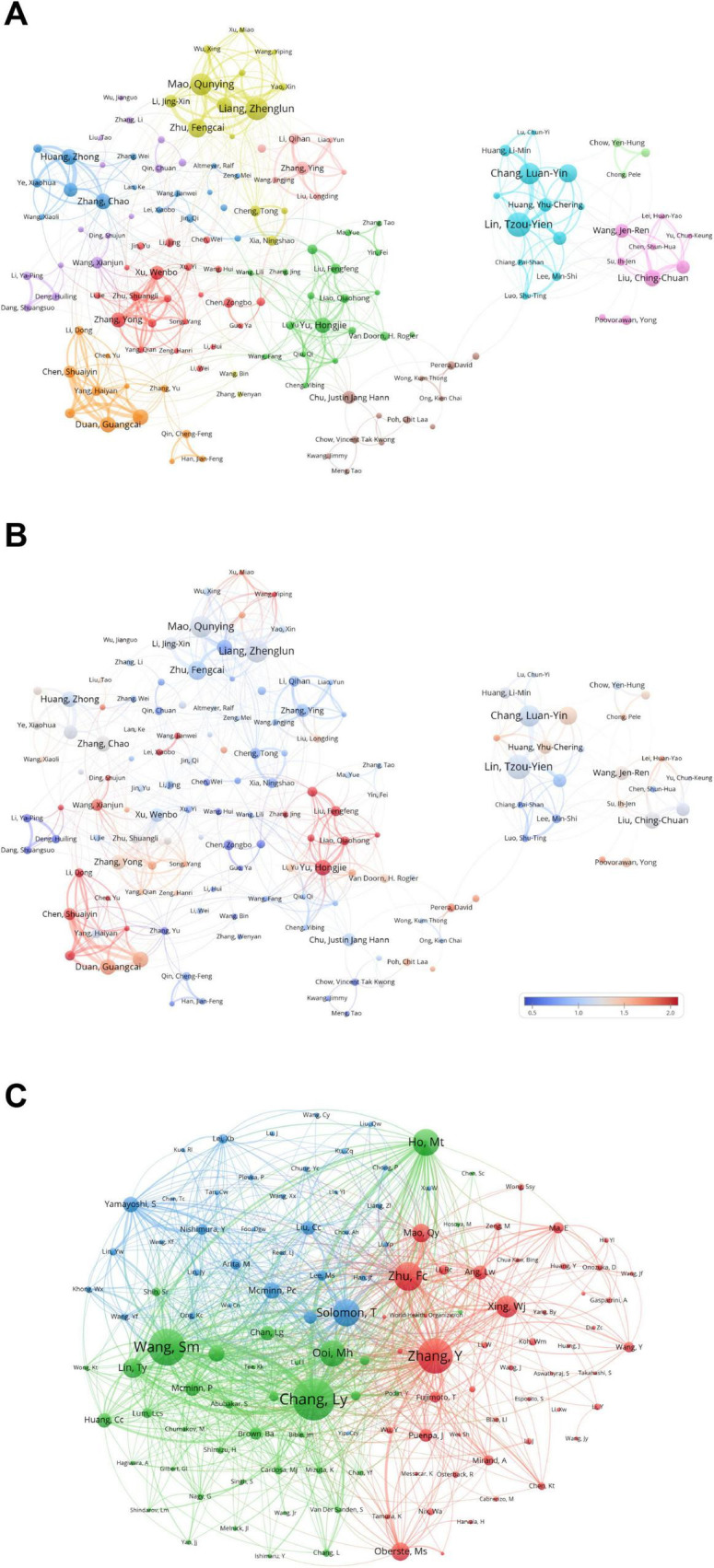
**(A)** This figure shows coauthors in the field of pediatric HFMD research, with node colors representing different clusters of authors. The node size indicates the frequency of cooccurrences, and the links indicate the relationships between cooccurring authors. **(B)** The graph indicates the strength of academic relationships, with a bluish node color indicating authors with fewer links and a reddish color indicating a higher density of links. **(C)** This graph shows the authors who are co-cited in studies related to pediatric HFMD, with node size indicating citation frequency. The visualization was produced via VosViewer and succinctly captures and analyzes the interconnections between cited authors in this research area.

The cooperative relationships among authors drawn by VOSviewer are presented in Figure 5A. On the basis of these cooperative relationships, authors in the research field of pediatric HFMD are divided into different clusters. The cyan, pink, and green clusters on the right side of the figure mainly include authors from the Taiwan Province of China, such as Lin, Tzou-Yien, Chang, Luan-Yin, Liu, and Ching-Chuan. Meanwhile, most of the authors on the left side of the figure are from mainland China, such as Liang, Zhenglun, Mao, Qunying, Zhu, Fengcai, Duan, and Guangcai. In addition, there are clusters representing Sino-foreign cooperation; for example, the brown cluster includes Chu, Justin Jang Hann, Perera, David, Meng, and Tao. Compared with the relatively independent clusters of authors from the Taiwan Province of China on the right side, the academic cooperative relationships among the clusters on the left side are more complex and diverse. Figure 5B shows that the small clusters with Yu, Hongjie, Liao, Qiaohong, Liu, Fengfeng, etc., as the core and those with Li, Dong, Chen, Shuaiyin, Chen, Yu, etc., as the core on the left side have the closest internal cooperative relationships.

The co-citation network among authors is shown as follows. Co-citation refers to the frequency with which two authors are simultaneously cited by a third-party author in the same literature. On the basis of this indirect citation relationship, the research relevance and article similarity among authors can be more intuitively revealed (26). In Figure 5C, the authors are divided into three different clusters according to the similarity of their research content. The red cluster, which is represented mainly by Zhang, Y, Zhu, Fc, Xing, and Wj, focuses on research on the application of biotechnology and applied microbiology in pediatric HFMD. Second, the green cluster, which includes authors such as Ooi, Mh, Ying-Chu Lin, Wang, and SM, is more inclined toward research in the immunology direction of this field. Finally, some authors who focus on virology, neuroscience, etc., are classified into the blue cluster, such as Solomon, T, McMinn, Pc, Liu, CC, etc.

### 3.5 Journal publication analysis

An analysis of 474 journals publishing research in this field was conducted to gain a better understanding of the current state of relevant publications. According to Table 4 and Supplementary Figure 2A, *Plos One* has the highest number of publications (117 articles), followed by *BMC Infectious Diseases* (64 articles), *Scientific Reports* (63 articles), and the *Journal of Medical Virology* (62 articles). *Journal of Virology* is the journal attracting the most attention, with a co-citation count of 3828 times, followed by *Plos One* (3332 times). Notably, among the top ten journals in terms of the number of published articles, nine are in the Q2 quartile or higher, with three in Q1. This feature is even more prominent in the co-citation ranking, where more than half of the journals are in Q1, including top-tier medical journals such as the *New England Journal of Medicine* (cited 1686 times, with an impact factor of 96.3). These findings indicate that the research trends and cutting-edge hotspots of pediatric HFMD have received extensive attention both within and outside the research field. Supplementary Figure 2B shows a correlation heatmap of journals. The heatmap visualizes the changes in the popularity of the journals that have made significant contributions to the research field of pediatric HFMD since 2000 through a time axis and classifies them according to the similarity of their research content (the dendrogram on the left). Before 2020, the research in this field was mostly confined to virology and epidemiology, corresponding to journals with higher popularity, such as the *Journal of Clinical Virology*, *Epidemiology and Infection*, *Pediatric Infectious Disease Journal*, etc. As time progressed, the research scope of this field expanded to broader and more diverse research fields, such as microbiology and public health, with journals such as the *International Journal of Environmental Research and Public Health*, *Frontiers in Microbiology*, *BMC Public Health*, etc.

**TABLE 4:** Ranking of the top ten major journals related to pediatric HFMD research from 2000 to 2025.

Rank	Journal	Publications	IF (JCR2023)	JCR quartile	Co-cited-journal	Co-citations	IF (JCR2023)	JCR quartile
1	*Plos One*	117	2.9	Q1	*Journal of Virology*	3,828	4.0	Q2
2	BMC Infectious Diseases	64	3.4	Q2	*Plos One*	3,332	2.9	Q1
3	Scientific Reports	63	3.8	Q1	Emerging Infectious Diseases	2,183	7.2	Q1
4	Journal of Medical Virology	62	6.8	Q1	Vaccine	1,783	4.5	Q2
5	Virology Journal	56	4.0	Q2	New England Journal of Medicine	1,686	96.3	Q1
6	*Journal of Virology*	54	4.0	Q2	Journal of Clinical Microbiology	1,638	6.1	Q1
7	Vaccine	53	4.5	Q2	Clinical Infectious Diseases	1,504	8.2	Q1
8	Archives of Virology	52	2.5	Q3	Journal of Clinical Virology	1,372	4.0	Q2
9	Viruses-Basel	38	3.8	Q2	Pediatric Infectious Disease Journal	1,277	2.9	Q2
10	Human Vaccines and Immunotherapeutics	37	4.1	Q2	Journal of Infectious Diseases	1,269	5.0	Q1

**FIGURE 6:**
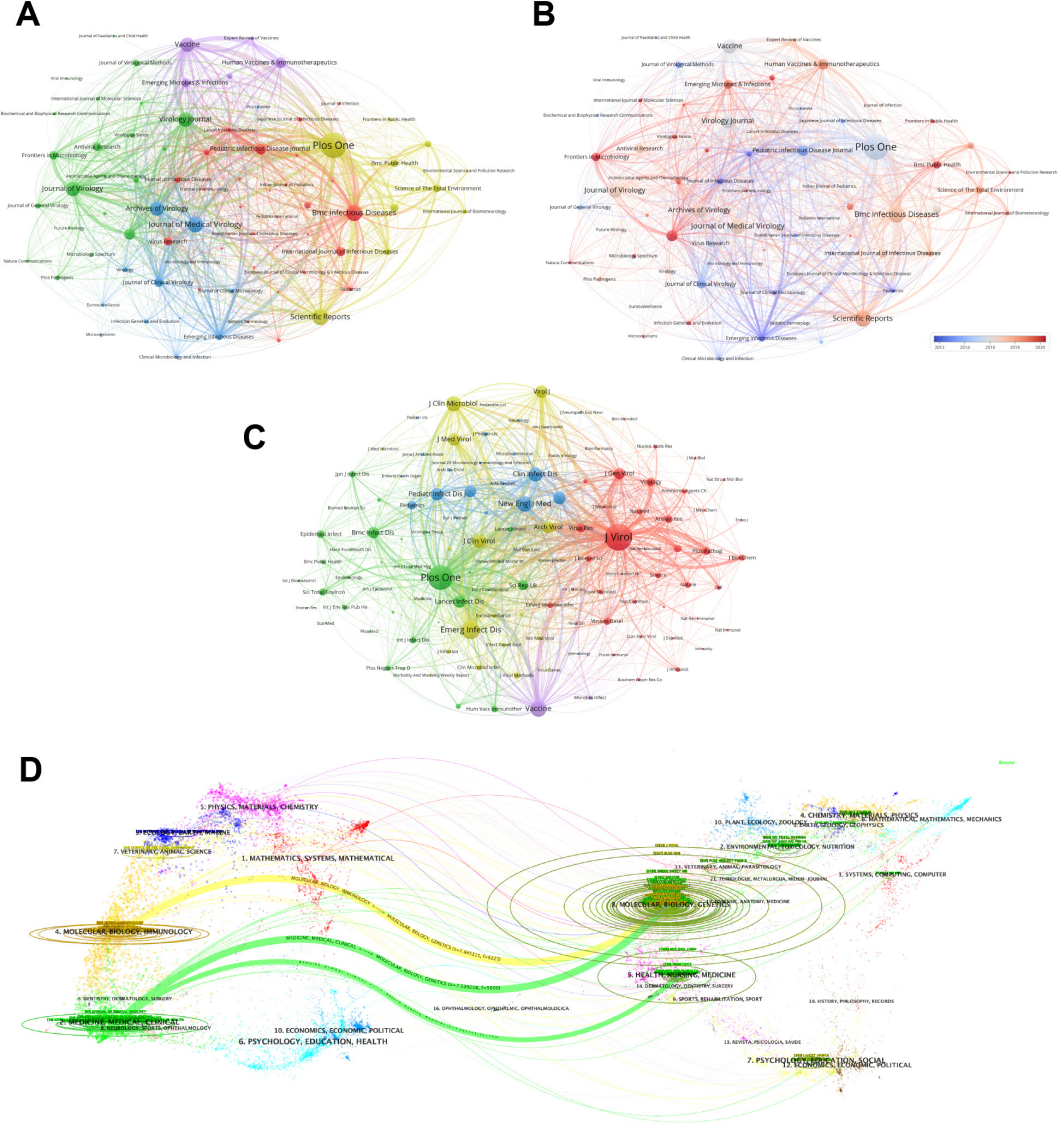
**(A)** Visualization and analysis of journal collaboration networks in VOSviewer. Journals in different clusters are distinguished by nodes of different colors, with node size representing their frequency of occurrence. **(B)** The time dimension is taken into account by adding to **(A)**, where red color indicates an increase in the impact of the field and blue color indicates a decrease in the activity of the field. Journals that have recently had a significant impact or have experienced a decrease in research participation are highlighted. **(C)** The figure depicts the co-citation relationships between research journals, with node size indicating the frequency of co-occurrences and connections indicating co-citation relationships. The node size reflects the importance and influence of the journal in the network. **(D)** Dual map visualizing journals related to pediatric HFMD research, with clusters of citing journals on the left and cited journals on the right. The colored tracks between them represent citation relationships.

The visualization results of the cooperative relationships among these journals are shown in Figure 6A. The yellow cluster includes journals focusing on research in the fields of public health, the environment, and health sciences (*e.g., Plos One*, *Scientific Reports*). The red cluster contains journals that focus mainly on infectious disease research (*Journal of Infection*, *Lancet Infectious Diseases*, etc.). Journals conducting in-depth basic research on virology are classified into the blue cluster (*Archives of Virology, Virology*, etc.). The green cluster, which is based on virology, further expands to include viral immunology, antiviral research, and molecular virology (*Journal of Virological Methods*, *Viral Immunology*, etc.). The last purple cluster is oriented toward vaccine development (*Vaccine*, *Expert Review of Vaccines*, etc.). As shown in Figure 6B, journals with relatively red dots, such as *Frontiers in Microbiology* and *BMC Public Health*, have been actively researched in this field in recent years. The co-citation relationships among journals are more intuitively presented in Figure 6C. These journals are briefly divided into five categories according to the similarity of research content reflected by the co-citation relationships between them. The red cluster includes journals focusing on basic virology research (*Journal of Virology*, *Journal of General Virology*, etc.). The green cluster covers most journals related to infectious diseases and public health (*Epidemiology and Infection*, *BMC Infectious Diseases*, etc.). Journals focusing on pediatric infectious diseases are classified into the blue cluster (*Pediatric Infectious Disease Journal*, *Journal of Pediatrics*, etc.). The yellow cluster involves mainly the fields of virology and infectious diseases (*Virology Journal*, *Journal of Medical Virology*, etc.). The last relatively small purple cluster includes journals focusing on the prevention and treatment of microbial infections (*Vaccine, Microbes and Infection*, etc.).

Figure 6D is a double-mapping overlay graph of journals, which can more intuitively present the citation relationships between various journals and the changes in research focus. The citation relationship is indicated by the citing journals on the left, with lines in the middle pointing to the cited journals on the right. The thicker yellow lines in the figure indicate that journals in the Molecular, Biology, Immunology directions mainly apply to the literature from journals in the Molecular, Biology, Genetics directions. Journals in the Medicine, Medical, and Clinical directions mostly cite literature from journals in the Molecular, Biology, Genetics and Health, Nursing, and Medicine directions.

### 3.6 Keyword analysis

As a guide to the research direction or a condensation of the research content of an article, keywords can usually highly summarize the main idea of the article. Therefore, keyword analysis is an important part of bibliometric analysis, which is highly important for understanding the current situation of a research field and predicting its development trend (27). The top 20 keywords in terms of occurrence frequency and total link strength are shown in Table 5. Among them, hfmd and enterovirus 71 are the core terms in this field, with occurrence frequencies of 698 and 575, respectively. Next are enterovirus (208 times) and coxsackievirus a16 (106 times).

**TABLE 5:** Ranking of the top twenty major keywords of pediatric HFMD research from 2000 to 2025.

Rank	Keyword	Occurrences	Total link strength	Rank	Keyword	Occurrences	Total link strength
1	HFMD	698	1,058	11	Coxsackievirus	31	68
2	Enterovirus 71	575	833	12	EV71 vaccine	31	56
3	Enterovirus	208	393	13	neutralizing antibody	31	62
4	Coxsackievirus a16	106	210	14	Meteorological factor	30	49
5	Children	97	174	15	Risk factor	30	61
6	Vaccine	80	186	16	VP1	30	66
7	Epidemiology	74	176	17	Encephalitis	28	63
8	Coxsackievirus a6	42	94	18	Molecular epidemiology	28	64
9	Immunogenicity	33	67	19	China	26	48
10	Phylogenetic analysis	33	67	20	Herpangina	26	62

The trend in the frequency of keywords over time from 20010 to 2024 is shown in Supplementary Figure 3A. The keywords “outbreak,” “disease,” “malaysia,” and other keywords emerged earlier and maintained their popularity for a long period of time. “hand,” “children,” “mouth-disease” and others were keywords that once received high attention in this field, whereas “associations,” “pollution” and “innate” have been popular keywords in recent years. In Supplementary Figure 3B, the popularity and development trends of the top 11 keyword clusters are compared horizontally and vertically in combination with the time axis. Each horizontal line represents a keyword group, with #0 being the largest cluster, and the size of the node on the horizontal axis is proportional to the co-citation frequency. For example, the earliest keyword in the largest cluster #0, “protein,” was “myocarditis,” and the “sequence” in #1, “aseptic meningitis,” and the “hand” in #2, “pulmonary edema,” also emerged relatively early. In contrast, #9 “pathological analysis” emerged relatively late, and “Chinese traditional medical” is a term that has attracted attention only recently. Notably, most of the keywords that emerged early in #2, “pulmonary edema,” had a relatively high co-citation frequency, indicating that this cluster might be a key research direction in the early stage of research on children’s hand, foot and mouth disease.

**FIGURE 7:**
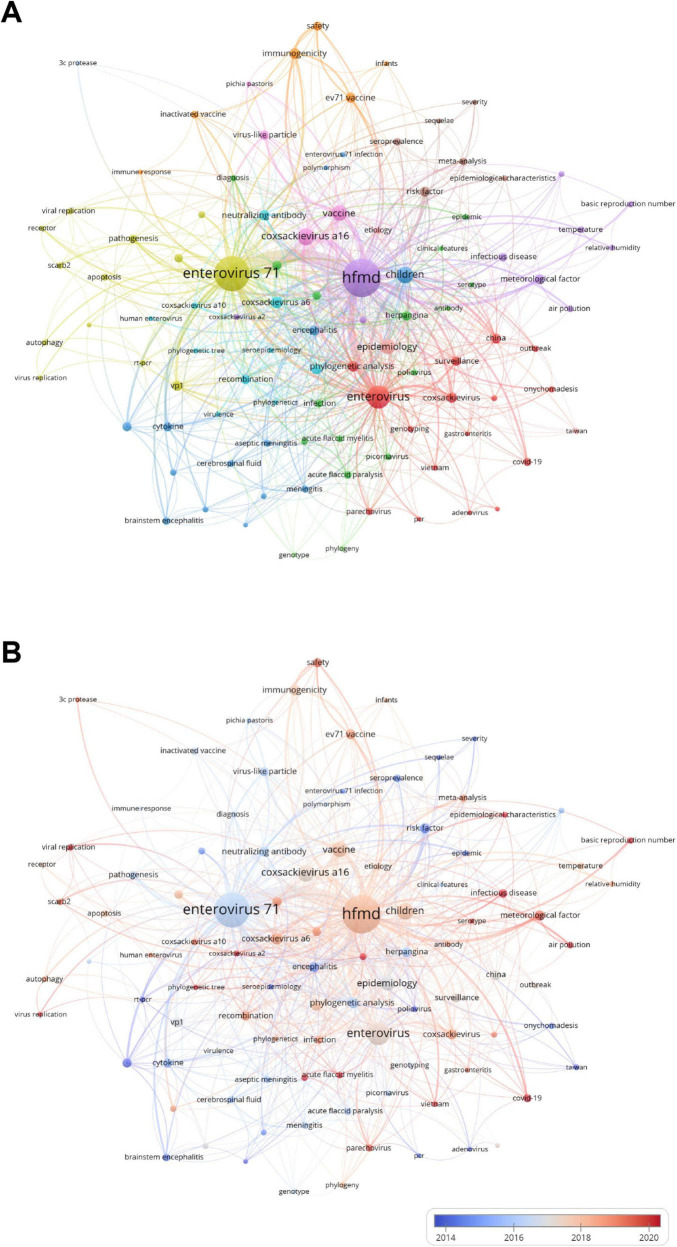
**(A)** Keyword map showing the links between keywords that have been studied in the field of pediatric HFMD research. The color-coded nodes represent different clusters of keywords. The node size indicates the frequency of co-occurrence, and the connections between nodes indicate the relationships between keywords. **(B)** This figure illustrates the relationship between each keyword’s recent contribution to research in the field and its overall output. Red indicates an increase in influence, and blue indicates a decrease in interest in the field. The color scale reflects the proportion of keywords over the past few years, highlighting terms that have had a significant impact or decreased engagement in this research.

The co-occurrence relationships among keywords related to pediatric HFMD are shown in Figures 7A,B. By analyzing these co-occurrence relationships, we can better understand the research directions and hot trends in this field. In the purple cluster of Figure 7A, “hfmd” with the highest co-citation count is the core keyword. In addition, there are terms such as “infectious disease,” “temperature”, “relative humidity,” “meteorological factor,” and “air pollution,” which focus on the impact of meteorological factors on infectious diseases. Second, the keywords related to the pathogenic mechanism of enterovirus 71 are classified into the yellow cluster, which includes “enterovirus 71,” “viral replication,” “receptor,” “scarb2,” etc. The blue cluster focuses on children as the main research direction, paying attention to children’s neurological diseases, and encompasses terms such as “children,” “encephalitis,” “aseptic meningitis,” “brainstem encephalitis,” etc. The red cluster includes keywords related to basic virology and research on multiple viruses, such as “enterovirus,” “coxsackievirus,” “phylogenic analysis,” “parechovirus.” The green cluster, interspersed among multiple clusters, mainly consists of terms for research on virus-related infectious diseases, such as “acute flaccid myelitis,” “acute flaccid paralysis,” “picornavirus,” etc. The cyan cluster includes “coxsackievirus a10,” “coxsackievirus a6,” “human enterovirus,” etc., and tends to research human enteroviruses, seroepidemiology, etc. The remaining smaller pink and orange clusters focus on vaccine research and development, and the brown cluster emphasizes epidemiological research. As shown in Figure 7B, keywords such as “infectious disease,” “air pollution,” and “acute flaccid myelitis” have received increased attention in recent years, which may be highly important for exploring the occurrence and development mechanisms of pediatric HFMD, intervention measures, and new research methods.

**FIGURE 8:**
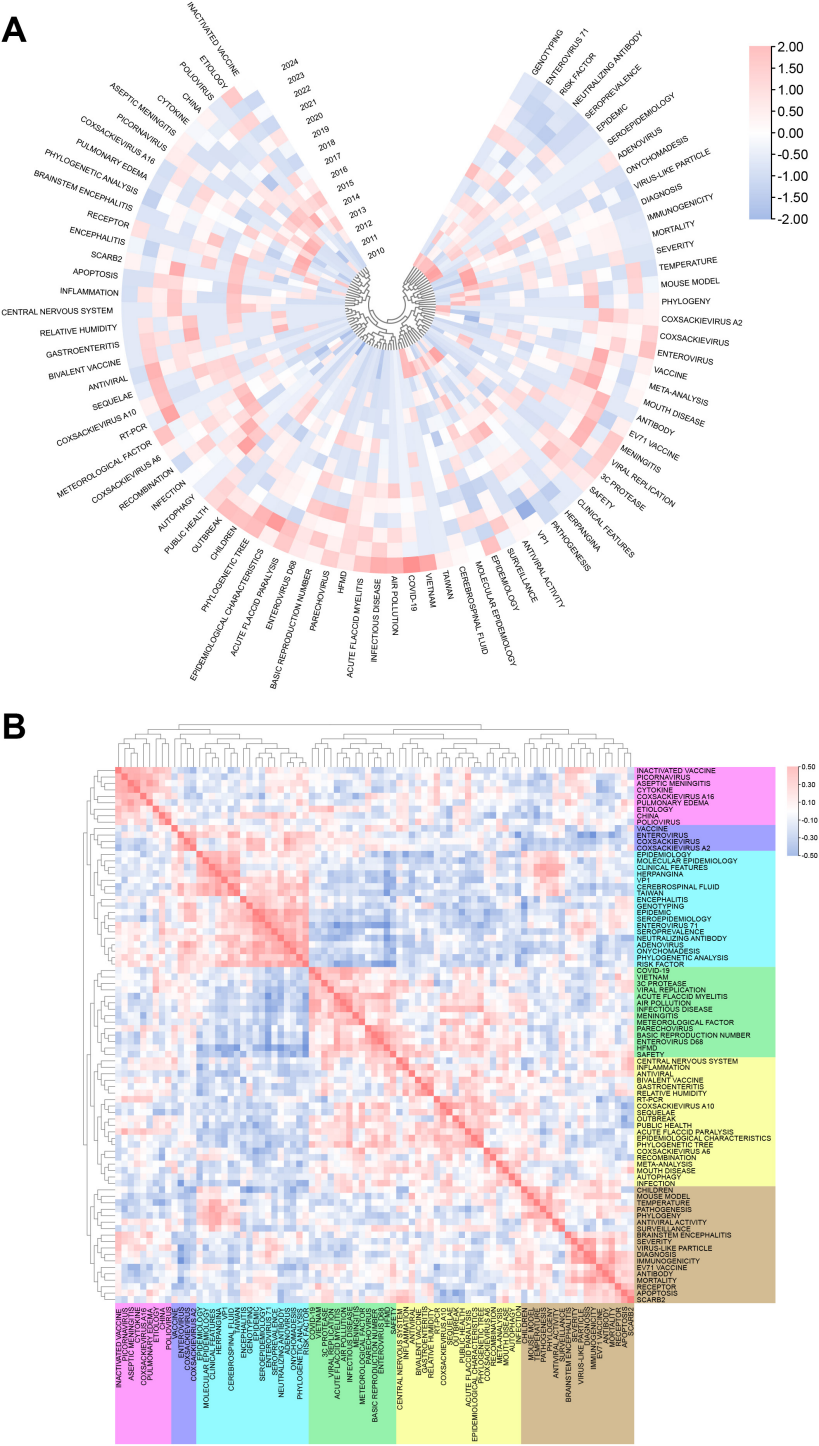
**(A)** The figure shows the correlation of keyword popularity, clustering keywords with similar peak periods of popularity together. **(B)** Heatmap showing the prevalence of keywords in the study of pediatric HFMD, divided into groups on the basis of the prevalence of the keywords in similar time periods and differentiated by color.

The heatmap in Figure 8A shows a more in-depth and detailed analysis of the evolutionary trends of the abovementioned research hotspots. Before 2020, research directions such as “scarb2,” “picornavirus,” and “immunogenicity” received much attention, but in recent years, their research popularity has declined. Moreover, terms such as “meningitis,” “mouth-disease”, and “autophagy” have gradually emerged. In the past 2 years, “phylogenetic tree,” “covid-19,” and “infectious disease” have become popular keywords. Figure 8B shows a heatmap analysis of keywords in the research field of pediatric HFMD. According to their correlation and popularity, they are divided into six main clusters. From top to bottom, the first cluster includes terms such as “coxsackievirus a16,” “poliovirus,” “picornavirus,” etc., which revolve around basic virology research. The cluster below is related to the research and development of enterovirus vaccines, which include “vaccine,” “enterovirus,” and “coxsackievirus.” The third cluster covers keywords such as “epidemiology,” “enterovirus 71,” “molecular epidemiology,” and “seroprevalence,” which focus on virology and epidemiology. Keywords concentrated in the fields of virology, infectious diseases, and public health, such as “viral replication,” “infectious diseases,” “basic reproduction number,” “public health,” and “relative humidity,” are classified into the fourth and fifth clusters. The last cluster mostly contains keywords related to medical research, such as “mouse model,” “temperature,” “surveillance,” and “mortality.”

**FIGURE 9:**
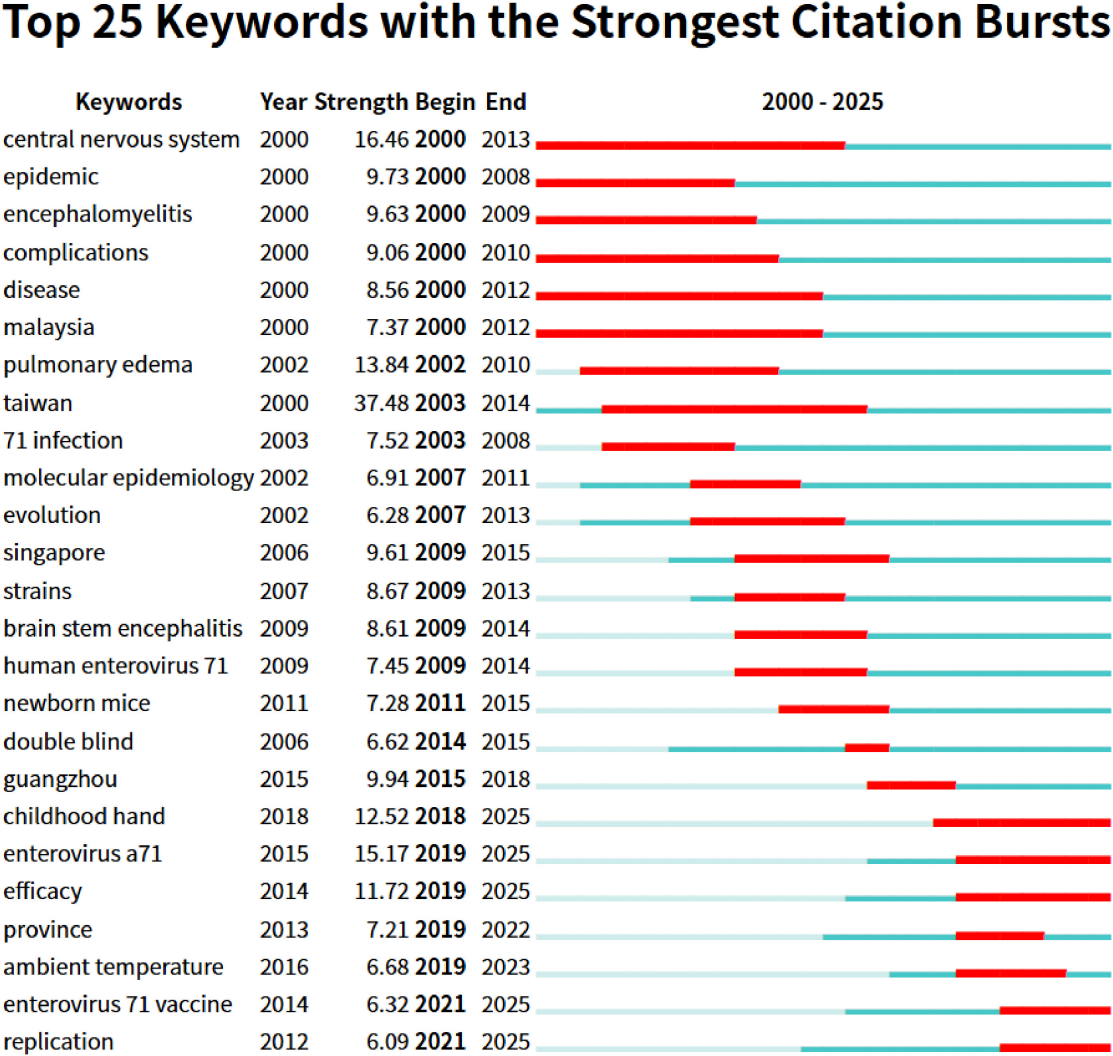
The diagram illustrates the 25 primary keywords characterized by pronounced bursts of citations, denoted by red spikes on the timeline. These bursts signify sudden surges in citation counts, signaling pivotal moments of emerging crucial questions or solutions within the field.

Figure 9 shows the top 25 keywords with the strongest citation bursts. It is easy to find that several keywords that started to burst in 2000 have relatively long burst periods. Among them, the burst period of the “central nervous system” extends from 2000 to 2013, which is the keyword with the longest burst period. Moreover, its burst strength is relatively high, reaching 16.46. Among all the keywords, the one with the highest burst strength is “taiwan,” which is as high as 37.48. The high level of attention given to this term is likely related to the high incidence of pediatric HFMD in Taiwan Province of China. Of particular note are the keywords at the bottom of the figure whose burst periods continue to the present, including “childhood hand” (burst strength 12.52), “enterovirus a71” (burst strength 15.17), “efficacy” (burst strength 11.72), “enterovirus 71 vaccine” (burst strength 6.32), and “replication” (burst strength 6.09). These terms reflect the current main research directions in this field to some extent.

### 3.7 Highly cited reference analysis

To some extent, the citation count of an article can indirectly reflect its quality and visually present the degree of attention and influence it receives within its field. Conducting an in-depth analysis of highly cited articles helps us efficiently and comprehensively grasp the research hotspots in the corresponding field. The basic information of the top fifteen articles in terms of citation count is shown in Table 6. The most highly regarded article is “Virology, epidemiology, pathogenesis, and control of enterovirus 71,” published by Solomon, T et al. in in *Lancet Infectious Diseases*. This literature reviews the virological characteristics, epidemiology, pathogenesis, and prevention and control measures of enterovirus 71. EV71 causes mainly HFMD and neurological complications in children. The Asia–Pacific region is an area with a high incidence. EV71 has diverse genetic subtypes and has evolved rapidly. Currently, prevention and control mainly rely on public health interventions, and vaccine research and development are underway (28).

**TABLE 6:** Ranking of the fifteen major highly cited references in pediatric HFMD research from 2000 to 2025.

Rank	Author	Article title	Source title	Cited	Year	Category	DOI
1	Solomon, T; Lewthwaite, P; Perera, D et al.	Virology, epidemiology, pathogenesis, and control of enterovirus 71	Lancet Infectious Diseases	1030	2010	Review	10.1016/S1473–3099(10)70194–8
2	Xing, WJ; Liao, QH; Viboud, C et al.	Hand, foot, and mouth disease in China, 2008–12: an epidemiological study	Lancet Infectious Diseases	719	2014	Article	10.1016/S1473–3099(13)70342-6
3	Ooi, MH; Wong, SC; Lewthwaite, P et al.	Clinical features, diagnosis, and management of enterovirus 71	Lancet Neurology	623	2010	Review	10.1016/S1474–4422(10)70209-X
4	Zhu, FC; Meng, FY; Li, JX et al.	Efficacy, safety, and immunology of an inactivated alum-adjuvant enterovirus 71 vaccine in children in China: a multicenter, randomized, double-blind, placebo-controlled, phase 3 trial	Lancet	438	2013	Article	10.1016/S0140–6736(13)61049–1
5	Chan, LG; Parashar, UD; Lye, MS et al.	Deaths of children during an outbreak of hand, foot, and mouth disease in Sarawak, Malaysia: Clinical and pathological characteristics of the disease	Clinical Infectious Diseases	435	2000	Article	10.1086/314032
6	Zhang, Y; Zhu, Z; Yang, WZ et al.	An emerging recombinant human enterovirus 71 responsible for the 2008 outbreak of Hand Foot and Mouth Disease in Fuyang city of China	Virology Journal	394	2010	Article	10.1186/1743–422X-7–94
7	Zhu, FC; Xu, WB; Xia, JL et al.	Efficacy, Safety, and Immunogenicity of an Enterovirus 71 Vaccine in China	New England Journal of Medicine	373	2014	Article	10.1056/NEJMoa1304923
8	Liu, YX; Wang, XJ; Liu, YX et al.	Detecting Spatial-Temporal Clusters of HFMD from 2007 to 2011 in Shandong Province, China	*Plos One*	373	2013	Article	10.1371/journal.pone.0063447
9	Wang, XX; Peng, W; Ren, JS et al.	A sensor-adaptor mechanism for enterovirus uncoating from structures of EV71	Nature Structural and Molecular Biology	334	2012	Article	10.1038/nsmb.2255
10	Nishimura, Y; Shimojima, M; Tano, Y et al.	Human P-selectin glycoprotein ligand-1 is a functional receptor for enterovirus 71	Nature Medicine	332	2009	Article	10.1038/nm.1961
11	Zhang, Y; Tan, XJ; Wang, HY et al.	An outbreak of hand, foot, and mouth disease associated with subgenotype C4 of human enterovirus 71 in Shandong, China	Journal of Clinical Virology	328	2009	Article	10.1016/j.jcv.2009.02.002
12	Chan, KP; Goh, KT; Chong, CY et al.	Epidemic hand, foot and mouth disease caused by human enterovirus 71, Singapore	Emerging Infectious Diseases	321	2003	Article	
13	McMinn, P; Stratov, I; Nagarajan, L; Davis, S	Neurological manifestations of enterovirus 71 infection in children during an outbreak of hand, foot, and mouth disease in Western Australia	Clinical Infectious Diseases	316	2001	Article	10.1086/318454
14	Ang, LW; Koh, BKW; Chan, KP et al.	Epidemiology and Control of Hand, Foot and Mouth Disease in Singapore, 2001–2007	Annals Academy of Medicine Singapore	313	2009	Article	
15	Van Tu, P; Thao, NTT; Perera, D et al.	Epidemiologic and virologic investigation of hand, foot, and mouth disease, Southern Vietnam, 2005	Emerging Infectious Diseases	303	2007	Article	10.3201/eid1311.070632

**FIGURE 10:**
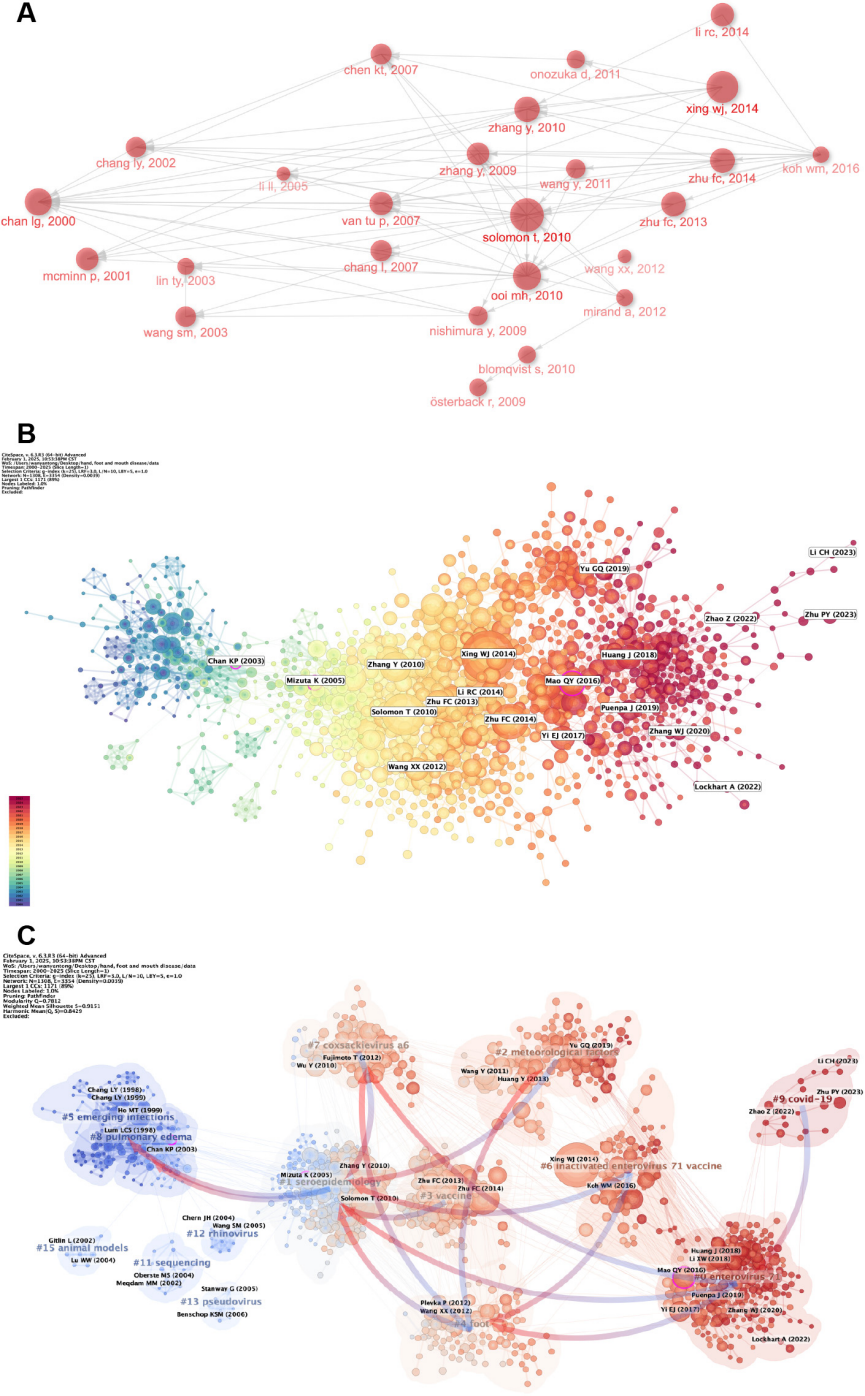
**(A)** The connectivity among the primary 25 citation bursts is demonstrated, depicting the citation interconnections among these articles with arrows. **(B)** Utilizing CiteSpace, a literature relation network diagram is generated. Various node colors represent different years, ranging from purple to red for later years. The size of each node corresponds to the frequency of the reference. **(C)** References are grouped on the basis of their likeness, where smaller numbers denote larger clusters, with #0 denoting the most substantial cluster. The node size reflects the frequency of co-citations, whereas the connections between nodes depict co-citation associations.

Figure 10A shows the co-citation relationship network diagram of highly cited studies. In this diagram, the paper “Deaths of children during an outbreak of hand, foot and mouth disease in Sarawak, Malaysia: Clinical and pathological characteristics of the disease” published by Chan, L G et al. in in *Clinical Infectious Disease* is particularly prominent (29). This paper laid an important foundation and provided key ideas for subsequent research in this field. During the development of this field, “Virology, epidemiology, pathogenesis, and control of enterovirus 71” published by Solomon, T et al. in in *Lancet Infectious Disease* (28) and “Clinical features, diagnosis, and management of enterovirus 71” published by Ooi, MH et al. in the same year in *Lancet Neurology* (30) played crucial bridging roles, promoting the gradual improvement of the knowledge system in this field. Finally, Koh, WM et al. comprehensively summarized and generalized previous studies, wrote “The Epidemiology of Hand, Foot and Mouth Disease in Asia A Systematic Review and Analysis,” and published it in *Pediatric Infectious Disease Jouarnal* in Koh et al. (31).

By analyzing the time–dimensional relationships among different studies on CiteSpace, as shown in Figure 10B, we can determine the publication times of highly cited articles and the potential relationships among them. Notably, the purple circles in the figure indicate that the contents or viewpoints of the articles might be important turning points in the development of this research field. The articles shown in the figure are “Epidemic hand, foot and mouth disease caused by human enterovirus 71, Singapore,” published by Chan, Kwai Peng et al. in in *Emerging Infectious Disease* (32); “Frequent importation of enterovirus 71 from surrounding countries into the local community of Yamagata, Japan, between 1998 and 2003,” published by Mizuta, K et al. in in the *Journal of Clinical Microbiology* (33); and “EV71 vaccine, a new tool to control outbreaks of hand, foot and mouth disease (HFMD),” published by Mao, QY et al. in in the *Expert Review of Vaccines* (34).

Figure 10C shows a visual representation of the potential correlations among these highly cited studies. We classified them into 16 different clusters according to their correlations. The largest cluster is enterovirus 71 #0, indicating that papers related to it have the highest number of citations. Next are #1 seroepidemiology and #2 meteorological factors, suggesting that seroepidemiology and meteorological factors remain key research directions in the field of hand, foot and mouth disease research in children. The arrows in the figure indicate the evolution of the article clusters. #9 COVID-19 has promoted the formation of enterovirus 71 #0, and enterovirus 71 has given rise to popular topics such as seroepidemiology #1, footing #4 and coxsackievirus a6 #7.

Supplementary Figure 4A presents the relationship between self-citations within the field and the total citation count of highly cited studies. For the paper “Risk factors for enterovirus 71 infection and associated HFMD/herpangina in children during an epidemic in Taiwan” published by Chang, LY et al. in in *Pediatrics* (35), its citations are mainly concentrated within the research field of pediatric HFMD. In contrast, studies with a lower LC/GC ratio have attracted widespread attention in the entire academic community. This means that the content of these studies has important reference value for multiple research directions. For example, “Human P-selectin glycoprotein ligand-1 is a functional receptor for enterovirus 71,” published by Nishimura, Y et al. in in *Nature Medicine* (36), and the research findings of Solomon, T et al. mentioned above (28), which ranks first in the number of citations.

Supplementary Figure 4B shows the citation burst situation. Among them, the article “Hand, foot and mouth disease in China, 2008–12: an epidemiological study” published by Xing, WJ et al. in in *Lancet Infectious Disease* is particularly notable (37). Although its burst period was short (from 2016 to 2019), its burst strength reached as high as 68.52, which fully demonstrates that its research content has attracted much attention in the academic community in a short period. In addition, the research of Ho Mt, Lum LCS, Huang CC and others had an early start of the burst period (38–40), playing a crucial role in promoting the development of early research in this field. Notably, for the article “The History of Enterovirus A71 Outbreaks and Molecular Epidemiology in the Asia-Pacific Region” published by Puenpa, J. et al. in in the *Journal of Biomedical Science* (41), its burst period continues to the present. This means that the research content and direction of this literature are likely to be the current focus of attention in this field.

## 4 Discussion

### 4.1 General distribution and global collaboration

This study employed bibliometric methods to conduct a systematic and comprehensive analysis of literature in the field of pediatric HFMD research from 2000 to early 2025. Data collection was completed on February 1, 2025. A total of 2,034 papers or reviews related to pediatric HFMD were included from 474 academic journals in the Web of Science Core Collection and PubMed. Research in this field involved 78 countries, 2,234 institutions, and 8,129 authors.

Based on annual publication output and citation trends, significant advancements in pediatric HFMD research were identified during the periods of 2009–2014 and 2017–2019 (Figure 2). These advancements include, but are not limited to: molecular epidemiological studies of pathogens (42); vaccine development and deployment (42–45); advances in rapid diagnostic technologies (46); exploration of antiviral therapies (47, 48); disease surveillance and public health interventions (49–51); and investigations into neurological complications and host–pathogen interactions (52–54). It is worth noting that the current body of research predominantly consists of descriptive or cross-sectional studies, covering areas such as epidemiological surveys, etiological analyses, clinical characterization, and vaccine development. A key limitation lies in the relative scarcity of longitudinal cohort studies. For instance, while various vaccines have demonstrated short-term effectiveness in reducing incidence rates, their long-term protective efficacy remains inadequately validated and requires further investigation across different age groups and immune statuses. Moreover, research efforts have been largely concentrated in East Asia, particularly China and Southeast Asia. There is a pressing need to expand studies to other regions with distinct circulating viral strains, such as areas dominated by Coxsackievirus A, to inform comprehensive global prevention and control strategies. Additionally, more rigorous clinical trial designs are essential. Future trials should be multi-center and large-scale, incorporating participants of diverse ages, socioeconomic backgrounds, and geographic locations to validate the generalizability and effectiveness of new interventions.

China dominates the research landscape in this field, contributing 71.63% of global paper output. The core group of authors and leading research institutions are also predominantly based in China (Tables 2, 3). This reveals the current uneven distribution of global research investment. Furthermore, analysis of global academic exchanges similarly exposes a significantly unbalanced pattern in scientific collaboration. Existing international cooperation networks are limited and primarily involve a handful of scientifically advanced countries, such as China, the United States, the United Kingdom, and Australia (Figures 3A,B). From the perspective of disciplinary development, establishing and strengthening extensive international academic exchange mechanisms is a prerequisite for advancing the field. In building these collaborative pathways, different economies can leverage their respective strengths for mutual benefit. As a key leader in South-South cooperation, China can deepen collaboration with neighboring developing countries through technology sharing (e.g., rapid HFMD diagnostic kit development), joint talent training programs, and collaborative research on regional transmission patterns. Developed countries like the United States and the United Kingdom can contribute on the resource supply side by providing specialized research funding, donating critical equipment, and sharing technical expertise such as epidemic surveillance models. Furthermore, leveraging transnational drug registration coordination mechanisms and technology transfer agreements can accelerate the global dissemination and application of high-quality research outcomes (55).

Another critical concern is the marginalization of low-and middle-income countries (LMICs) with high HFMD disease burdens but low research output, such as Pakistan and Indonesia. Challenges such as insufficient research funding, underdeveloped public health systems, shortages of research equipment and trained personnel, weak infrastructure (including transportation and communications), and inadequate accuracy and timeliness of surveillance data hinder their integration into core collaborative networks. In this context, international support from diverse stakeholders is crucial to address these disparities. Specifically: At the international organization level, the World Health Organization (WHO) can support LMICs through transnational capacity-building initiatives, assisting in the establishment of standardized viral specimen banks and genomic databases. Laboratory quality certification programs under frameworks like the Global Health Security Agenda (GHSA) can enhance laboratory testing capabilities and data reliability in LMICs (56). Regionally, cooperation frameworks such as the Association of Southeast Asian Nations (ASEAN) public health collaboration can foster synergy by integrating regional resources, enabling shared laboratory facilities, joint human resource training, and pooled funding mechanisms. Furthermore, active global researcher participation in initiatives like the Open Science Framework (OSF) and the Global Health Data Exchange (GHDx) should be encouraged (57). By adhering to standards for data security and intellectual property protection, these platforms can significantly enhance the accessibility and usability of relevant data.

### 4.2 Development, hotspots, and frontiers

Analyzing keywords in the research fields of pediatric HFMD can help us better grasp the frontiers and hotspots of research in this area. The main keywords used in current research include “hfmd, enterovirus 71,” “enterovirus,” “coxsackievirus a16,” “children,” “vaccine,” “epidemiology,” etc. These keywords highlight popular topics in the research fields of pediatric HFMD. On the basis of the cluster and heatmap analysis of keywords (Figures 7, 8) and the cluster analysis of highly cited literature (Figure 10C), research related to pediatric HFMD has focused on four main directions: research on pathogenic mechanisms, epidemiological research, novel therapeutic discoveries and vaccine development.

#### 4.2.1 Pathogenic mechanisms

In 1969, the EV71 virus was first isolated from fecal specimens of infants with central nervous system diseases in California, USA. Through cell culture and serological testing, researchers determined that while its morphology resembled known enteroviruses, its biological characteristics were distinct. This led to its official classification as a new enterovirus by the International Committee on Taxonomy of Viruses in 1979 (58). This initial research phase was predominantly dedicated to pathogen identification. Viruses such as CVA16 and EV71—both members of the Picornaviridae family—were recognized early on. However, EV71 rapidly became the primary research focus due to its strong propensity to cause severe neurological complications, such as brainstem encephalitis. Epidemiological studies established the generally self-limiting nature of HFMD, which typically resolves without sequelae. Nonetheless, recurrent outbreaks of EV71 in the Western Pacific Region prompted a swift research shift toward severe cases. This reorientation inadvertently reduced the representativeness of other strains like CVA16 in research agendas, despite CVA16 being identified early as a common causative agent (59, 60). In recent years, studies have continued to affirm the polyviral etiology of HFMD, emphasizing that the disease is caused by multiple enteroviruses, including CVA6, CVA16, CVA10, and EV71. However, research investment into their respective pathogenic mechanisms remains highly uneven. For example, the introduction of an EV71 vaccine in China in 2016 further intensified focus on this particular strain. In contrast, the molecular mechanisms of other strains like CVA6 and CVA10—whose contribution to major HFMD outbreaks has been increasingly significant—have been comparatively underexplored (61–63). This imbalance can be partially attributed to the inertia of early research priorities, which has perpetuated the underrepresentation of non-EV71 strains in mechanistic studies.

As evidence of EV71’s role in severe HFMD accumulated, research progressed into an animal model phase aimed at validating infection mechanisms. Early animal models, primarily using mice, were employed to understand EV71’s neuroinvasive potential, including how it invades the central nervous system via the neuromuscular junction and leads to fatal neurological complications. These models were initially developed largely for EV71, driven by its association with severe disease phenotypes such as encephalitis and pulmonary edema. In comparison, research models for other strains like CVA16 and CVA6 were less developed and often faced limitations in fully recapitulating the diversity of human clinical manifestations (64, 65). This disparity further amplified the research emphasis on EV71 and overlooked the emerging importance of CVA6 and CVA10 in outbreaks—where CVA6, for instance, has frequently been identified as a predominant pathogen. Recent animal model research has attempted to correct this imbalance by expanding to include multiple strains and refining model systems. For instance, while mouse models have been used to assess long-term sequelae of EV71 infection, they have also highlighted the significant roles CVA16 and CVA6 play in outbreaks—roles for which corresponding animal experiments to elucidate their specific mechanisms are still lacking. Some recent efforts have aimed to develop more human-relevant models to reduce reliance on traditional animal studies, but these initiatives remain predominantly centered on EV71. A case in point is a study utilizing a mouse model to test an EV71-targeting therapeutic peptide, which overlooked similar mechanistic explorations for other strains, thereby underscoring the persistent underrepresentation of CVA10 and CVA6 in experimental research (65, 66).

The mainstream research direction subsequently shifted toward molecular and genomic exploration, focusing intently on molecular mechanisms and genetic variation. Early molecular investigations concentrated on EV71’s characteristics as a single-stranded RNA virus, including its high mutation rate attributable to an error-prone RNA polymerase—which generates quasispecies and haplotype diversity—and how these mutations enhance viral virulence, particularly in neuroinvasion. This depth of investigation was especially pronounced for EV71, partly because circulating variants like the C4 subgenogroup in the Western Pacific Region became priority vaccine targets. Conversely, the molecular mechanisms of other strains, such as capsid loop structures in CVA16, received comparatively less attention (67–69). Recent molecular and genomic studies have substantially enriched our mechanistic understanding. For EV71, research has delved deeply into specific mechanisms, such as the dual role of the tribbles pseudokinase 3 (TRIB3) pseudokinase in promoting infection, antagonism of interferon signaling pathways, and how capsid protein mutations influence viral entry, alongside advancing antiviral target development, including the monoclonal antibody E1 (70–72). However, similar mechanistic studies for other strains like CVA6 and CVA10 remain limited. Despite CVA6 and CVA10 being key pathogens in HFMD outbreaks—particularly in recent epidemics in China—research on their molecular mechanisms, such as the impact of genetic recombination on pathogenicity, is notably underrepresented. For example, while a chimeric virus study attempted to compare the capsid loops of EV71 and CVA16, similar efforts have not been extended to CVA6 and CVA10, thereby constraining the development of broad-spectrum therapies effective against multiple co-circulating strains (69, 73, 74).

In summary, the historical evolution of HFMD pathogen research demonstrates a clear progression from pathogen identification, through animal model validation, to in-depth molecular exploration. However, the field has been predominantly shaped by an intensive focus on EV71, leading to significant imbalances in research scope and resource allocation. Although recent studies have begun to acknowledge the important roles of CVA6, CVA16, and CVA10 in outbreaks—particularly in the post-vaccine era where the prevalence of these non-EV71 strains has increased—their representation in mechanistic investigations remains inadequate. For instance, while molecular target identification and vaccine development for EV71 are well-established (75, 76), research on genetic recombination and pathogenic mechanisms of CVA6 and related strains is relatively scarce (63, 77). Future research must strive for a more balanced and comprehensive exploration of multiple enterovirus strains to effectively address the complex and dynamically evolving epidemiological landscape of HFMD.

#### 4.2.2 Epidemiological studies

Asia is one of the regions with the highest incidence of HFMD, particularly in East and Southeast Asian countries, where the primary pathogenic strains are EV71 and CVA16. In Southeast Asia, multiple nationwide surveillance studies indicate that countries such as China, Vietnam, and India experience seasonal outbreaks (mostly occurring from late spring to early summer) nearly every year. Preschool children, especially those under 3 years old, constitute the most affected population (78–80). In China, the epidemiological characteristics of HFMD have undergone significant changes since the introduction of the EV71 vaccine in 2016. Both the incidence and severity rates associated with EV71 have markedly decreased. Meanwhile, the prevalence of strains such as CVA6, CVA10, and CVA16 has shown an upward trend across different regions (2, 81–83). In Europe, outbreaks of hand, foot, and mouth disease are relatively uncommon and primarily occur among young children. Compared to Asia, severe cases and mortality rates are lower. This may be related to differences in the predominant viral strains. Reports indicate that CVA6, CVA10, and CVA16 are the primary causative strains of hand, foot, and mouth disease in Europe (84–87). Reports of severe outbreaks dominated by EV71 are relatively scarce. The situation in the Americas and Oceania mirrors that in Europe, with relatively infrequent HFMD epidemics. However, outbreaks caused by CVA6 appear more prevalent (88–91). A few severe outbreaks caused by EV71 have also been reported (92). Relevant reports from Africa are scarce. A study in Tunisia reported a Tunisian CVA24 strain with a high degree of sequence difference in the VP1 coding region compared with other CVA24 strains. This is the first reported CVA24 strain that causes aseptic meningitis (93).

Regarding risk factors influencing the spread of HFMD, meteorological factors, air pollution, and behavioral factors have been extensively discussed by scholars. Among meteorological factors, relative humidity and temperature play a dominant role. Both relatively low and high humidity levels appear to be associated with increased HFMD incidence rates. A multicity study in mainland China revealed that the relationship between relative humidity and HFMD incidence approximates a U-shaped curve. The relative risk is the lowest when the relative humidity is 45%, and the highest when it is 20% or exceeds 85%. Moreover, there is spatial heterogeneity in this relationship (94). A study in the Sichuan Basin revealed that the relationship between relative humidity and the incidence of HFMD is J shaped. When the relative humidity exceeds 70%, the risk increases with increasing humidity, and the infection burden affected by high humidity is greater in the southern part of the basin (95). On the other hand, both low and high temperatures increase the risk of hand, foot, and mouth disease. The cumulative effect of high temperatures peaks at a lag of 0–10 days, while the cumulative effect of low temperatures peaks at a lag of 0–3 days (96). In Wuhan, there is a non-linear “M”-shaped relationship between temperature and the incidence of HFMD, with two peaks (4). In Shandong, the analysis of data from 2015 to 2019 via the multiscale geographically and temporally weighted regression (MGTWR) model revealed that temperature and humidity were positively correlated with the incidence of HFMD in spring and summer (97). Pearson, Dharshani et al., on the basis of relevant data from California, noted that in both the cold and warm seasons, an increase in temperature is associated with an increased risk of emergency department visits for HFMD. In coastal areas, the association is stronger in the cold season, possibly because mild and humid winter conditions are more conducive to the survival of pathogens (98).Additionally, factors such as daily sunshine duration, precipitation, and wind speed may also be associated with HFMD incidence (99).

In terms of air pollution, a study in Fuyang revealed that temperature and PM2.5 are the main risk factors for HFMD, and there is a synergistic effect between PM and meteorological factors. For example, the relative risk (RR) values related to the association between PM2.5 and HFMD vary significantly among different temperature groups. Children under 5 years of age, especially infants aged zero to 1 year, are more sensitive to environmental variables (100). An earlier study revealed that short-term exposure to PM2.5 and its components (especially black carbon, sulfate, ammonium, nitrate, and soil dust) is significantly associated with prolonged hospital stays in HFMD patients (101). A study in Zhejiang Province analyzed county-level data from 2013 to 2021 via a Bayesian spatiotemporal model. An increase in the concentrations of PM10 and NO_2_ is significantly associated with an increased risk of HFMD, whereas O_3_, SO_2_, and CO are negatively correlated. Meteorological factors significantly affect the relationship between air pollutants and the incidence of HFMD, and the association is more significant under extreme weather conditions (102).

The primary mode of HFMD transmission is the fecal-oral route. Additional transmission pathways include respiratory spread—through inhalation of droplets from coughing or sneezing, or contact with airborne particles contaminated by patient secretions—and direct contact transmission, which involves exposure to patients’ nasal or oral secretions, skin blister fluid, feces, or contact with contaminated objects such as toys or clothing. Regarding behavioral factors influencing pediatric HFMD spread, Kindergarten size and class structure are significant behavioral transmission factors. Studies indicate that reducing class size substantially lowers HFMD transmission risk, with incidence increasing by approximately 11% for every 10-person increase in class size. Large-scale kindergartens (> 300 children) carry a 40% higher transmission risk than small-scale ones (< 150 children), directly linked to the frequency of close contact among children. Inter-class interactions (e.g., shared activity rooms) also accelerate cross-class transmission (103). Furthermore, poor personal and public hygiene practices—such as inadequate hand hygiene and insufficient disinfection of tableware—can also substantially increase the risk of infection (104). Vaccination represents a key intervention for HFMD prevention, particularly in high-risk regions. The administration of EV71 vaccines has been demonstrated to effectively reduce infection rates. However, vaccine dissemination remains influenced by regional development disparities and variations in public health education (105).

#### 4.2.3 Novel therapeutic discoveries

The early symptoms of HFMD typically include fever, skin rash—commonly appearing on the hands, feet, and oral cavity—as well as oral herpes or ulcers. These manifestations are generally self-limiting and resolve spontaneously in children (2, 106). However, in severe cases, HFMD can lead to serious neurological complications and even mortality (107, 108). Current clinical management relies predominantly on supportive care—aimed at alleviating symptoms—due to the absence of specific antiviral therapies. Available vaccines, such as inactivated EV71 vaccines, offer limited protection, primarily targeting specific strains (61, 109, 110) and failing to cover emerging or atypical viral variants (60, 62). Consequently, there is a pressing need to develop novel therapeutic strategies.

Mechanism-based treatments targeting pathogen infection represent a prominent research direction, encompassing viral entry inhibitors and immunomodulatory approaches. In the realm of viral entry inhibition, certain molecules can block virus attachment to host cells. For instance, tannic acid derivatives such as chebulagic acid and punicalagin have been identified as potent broad-spectrum entry inhibitors effective against multiple viruses—including HFMD-associated pathogens—that utilize cell surface glycosaminoglycans (111). Furthermore, the mechanism of EV71 entry into human oral cells has been shown to be independent of classical pathways such as clathrin or caveolin, providing a foundation for designing specific inhibitors (112). Monoclonal antibodies targeting CVA16, including 9B5 and 8C4, have also been characterized as effective entry inhibitors capable of neutralizing the virus and reducing infection risk (113). Regarding immunomodulation, strategies aimed at regulating host immune responses to control infection severity are increasingly important. For example, toll-like receptor 7 (TLR7) recognizes viral RNA and plays a role in EV71 infection (114), and specific TLR polymorphisms are associated with HFMD severity, suggesting potential immunomodulatory targets (115). The interferon (IFN) signaling pathway is crucial in controlling EV71 and CVA16 infections; interferon-stimulated genes such as Cholesterol 25-hydroxylase (CH25H) can effectively block replication of these viruses (70). Other mechanisms include the role of Ragulator proteins in mediating EV71-induced apoptosis and pyroptosis, and identifying such molecules provides a basis for developing immunomodulatory therapies (116). Additionally, macrophage-mediated immune dysregulation may contribute to severe HFMD, supporting intervention through modulation of inflammatory responses (117).

Emerging therapeutic approaches are also focusing on novel molecular targets and alternative strategies, including RNA-based interventions and host-directed antivirals. In RNA-targeted therapy, methods such as siRNA screening have been employed to explore EV71 entry and replication mechanisms, identifying specific genes involved in membrane trafficking as potential targets (118). RNA methylation (m6A) modifications have been found to regulate host responses to EV71, offering intervention opportunities by influencing viral replication and immune pathways (119). Moreover, mRNA vaccines are under development as a novel platform targeting non-enveloped viruses like EV-A71 (120). In the domain of alternative therapies and molecular targets, various host-directed molecules have shown promise as antiviral agents. For example, a compound named 14S-(2’-chloro-4’-nitrophenoxy)-8R/S, 17-epoxy andrographolide has demonstrated efficacy in inhibiting EV-A71 infection (121). Fucosylated chondroitin sulfates extracted from sea cucumbers also exhibit antiviral activity (122). Other alternative pathways, such as modulation of host genes via inflammasomes or prostaglandin E2 regulation, offer new therapeutic avenues (123). Although these studies primarily target other viruses, their underlying mechanisms may be applicable to HFMD. However, most of these findings remain at the research stage and require further validation and clinical translation.

#### 4.2.4 Vaccine development

The development of vaccines for HFMD is an ongoing process that must contend with a complex and evolving pathogen landscape. To better control enterovirus diseases like HFMD, the Asia-Pacific Network for Enterovirus Surveillance (APNES) was established in 2017. Its mission is to assess the burden of enterovirus diseases, understand viral evolution, and promote the development of corresponding vaccines by coordinating laboratory diagnostics and data collection (124). APNES operates as a practical “surveillance-to-vaccine” model, emphasizing that an effective vaccination program relies not only on the vaccine itself but also on a robust and continuous epidemiological and virological surveillance system. This framework is crucial for controlling diseases like HFMD, which are caused by multiple, constantly evolving viruses.

In the initial phase of vaccine development, efforts were predominantly focused on creating monovalent vaccines targeting EV71, due to its strong association with severe neurological complications and fatalities (60, 62, 107). A key milestone in this phase was the approval and launch of three inactivated EV71 vaccines in China in 2016. Clinical trials confirmed these vaccines were highly effective, demonstrating a vaccine efficacy (VE) exceeding 90% against EV71-associated HFMD and a favorable safety profile (61, 125). Subsequent real-world evidence further validated their public health value, showing that a two-dose regimen significantly reduced EV71-associated hospitalizations and mortality in pediatric populations (125, 126).

However, the widespread administration of EV71 vaccines led to a significant shift in the HFMD epidemiological landscape. Non-EV71 enteroviruses, such as CVA6, CVA10, and CVA16, have increasingly become the dominant pathogens in outbreaks (62, 77, 127). This shift exposed a fundamental limitation of monovalent vaccines: their inability to provide cross-protection against multiple pathogenic serotypes. Consequently, the focus of vaccine R&D has pivoted toward developing multivalent formulations aimed at achieving broader protection from a single vaccine. Currently, bivalent, trivalent, and even quadrivalent vaccine candidates targeting combinations of EV71, CVA16, CVA10, and CVA6 are under active investigation (128, 129). For instance, a trivalent inactivated vaccine targeting EV71, CVA16, and CVA10 has demonstrated broad passive protection against multiple viral challenges in mouse models (60). Furthermore, novel platforms like virus-like particles (VLPs), which mimic the native virus structure without containing genetic material, are considered ideal candidates for constructing these multivalent vaccines (130, 131). The goal is to provide cross-serotype protection via a single preparation, enabling more comprehensive control of HFMD (110, 131).

Despite this progress, several challenges persist in current vaccine development. First, strain coverage remains limited. Most existing multivalent vaccine candidates are still in experimental stages, and their efficacy in humans requires further validation and clinical translation (61, 109, 110). Second, continuous viral evolution poses a long-term threat. The high variability of enteroviruses, prone to genetic recombination and mutation, can lead to the emergence of novel variants capable of evading existing immunity. For example, CVA6, an emerging pathogen, is undergoing rapid evolution, which could compromise vaccine effectiveness and alter outbreak patterns (109). Concurrently, promising candidates like VLPs face stability challenges, such as a potential rapid decline in immunogenicity at high concentrations, adding complexity to multivalent design (130). These collective challenges underscore the urgent need for multivalent and adaptable vaccine platforms. On one hand, developing multivalent vaccines capable of covering currently dominant and emerging strains is considered a key strategy for effective HFMD management (110, 126, 129, 132). On the other hand, it is imperative to leverage highly adaptable technological platforms—such as VLPs and mRNA vaccines—to enable rapid responses to viral evolution (120, 130, 131). Future vaccine design must therefore incorporate more advanced approaches, such as optimizing VLP stability or exploring rapidly adaptable mRNA platforms, to enhance both the breadth and durability of vaccine-induced protection.

### 4.3 Strengths and limitations

This study employs bibliometric methods to systematically review research on pediatric HFMD from 2000 to 2025, based on data from the WOSCC and PubMed. Using analytical tools such as CiteSpace, VOSviewer, and R-bibliometrix, an in-depth analysis of relevant literature was conducted across multiple dimensions, including publication volume, citation frequency, geographical distribution, contributions by authors and institutions, journal preferences, keywords, and cited references. The aim is to comprehensively present the developmental trends and research hotspots in this field. Compared to a bibliometric study on HFMD published in 2024 (133), this research covers a longer time span and integrates two major databases—WOSCC and PubMed—thereby enhancing the comprehensiveness and timeliness of the data. Through more refined visual and analytical approaches, this study identifies current research priorities in pediatric HFMD, examines the distribution of scholarly efforts, and highlights under-explored areas. It also systematically reveals China’s dominant role in global research and its patterns of international collaboration, while addressing issues such as the marginalization of low-income, high-burden countries within research networks. Furthermore, by synthesizing a wide range of recent studies, this paper provides a detailed exploration of four key research directions: pathogenic mechanisms, epidemiology, novel therapeutic strategies, and vaccine development. The findings offer more comprehensive and forward-looking bibliometric evidence to inform global strategies for the prevention and control of hand, foot, and mouth disease.

However, several limitations inherent to the bibliometric approach should be considered. Firstly, the restriction to English-language publications introduces potential language bias, as significant research published in other languages may have been overlooked. Secondly, the reliance solely on the WOSCC and PubMed, despite their prominence, means that studies indexed in other regional or specialized databases were excluded, limiting the comprehensiveness of the dataset. Furthermore, the temporal scope was constrained to literature published from 2000 onward due to database accessibility, thus omitting potentially influential earlier works. The cutoff date for data collection (February 1, 2025) also results in incomplete coverage of publications from the full year. These common bibliometric constraints—language, database coverage, and temporal boundaries—may affect the generalizability of the findings. Future research could benefit from incorporating multilingual literature and expanding database sources to improve the representativeness and depth of analysis.

## 5 Conclusion

This bibliometric analysis comprehensively presents the development trends and research hotspots in the field of pediatric hand-foot-and-mouth disease by deeply examining 2,034 relevant papers published since the 21 st century (up to February 2025). It highlights China’s leading contributions in this research domain while reflecting the global focus on its pathogenesis, epidemiology, novel therapeutic discoveries, and vaccine development. However, critical research gaps persist: excessive focus on EV71 at the expense of other highly prevalent serotypes like CVA6 and CVA10; insufficient international collaboration—particularly with low-income countries bearing high disease burdens; and inadequate coverage of multivalent vaccines. Future research should prioritize establishing equitable global partnerships, deepening comprehensive studies on multiple enterovirus serotypes, and accelerating the development of broad-spectrum vaccines and targeted antiviral therapies to achieve effective global prevention and control of HFMD.

## Data Availability

The original contributions presented in this study are included in this article/Supplementary material, further inquiries can be directed to the corresponding author.
