# Supplementary material for: Historical evolution, research hotspots and emerging trends of pediatric hand, foot, and mouth disease: a bibliometric worldview since the 21st century
Source: Front Med (Lausanne). 2025 Dec 5;12:1722750. doi: 10.3389/fmed.2025.1722750 (PMC12714650; doi:10.3389/fmed.2025.1722750)
Supplement: Supplementary file 1 [file Data_Sheet_1.docx]

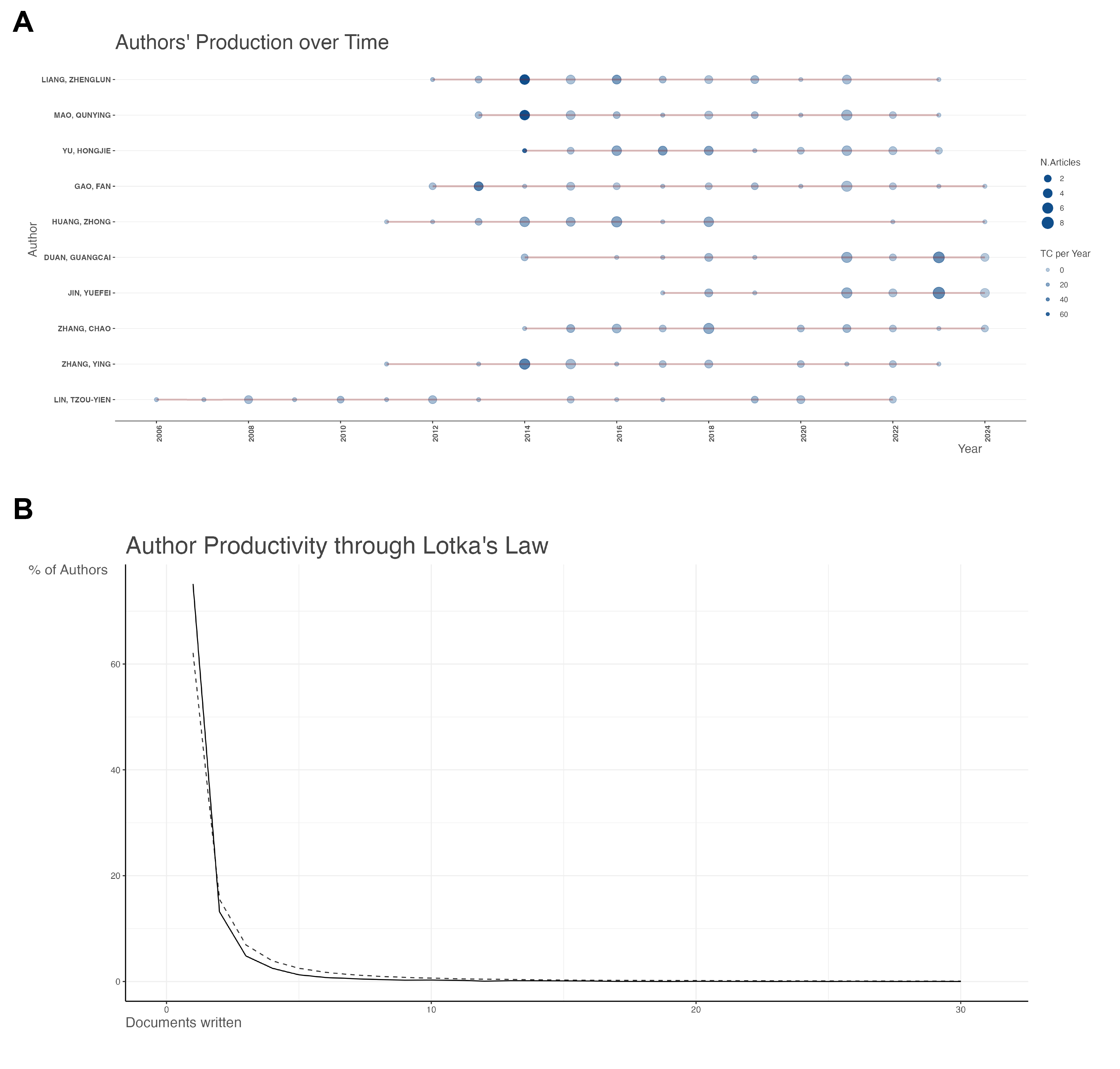


**Supplement Figure 1**

**(A)** The visual analysis depicts the publications of the top ten authors ranked by frequency of appearance from 2005 to 2025. In the visual analysis, the length of the line indicates the duration of sustained publications, the size of the dots indicates the number of papers in chronological order, and the color of the dots indicates the frequency of citations. **(B)** Authors' output analysis for Lotka's Law, with dashed lines showing ideal values and solid lines showing actual values.


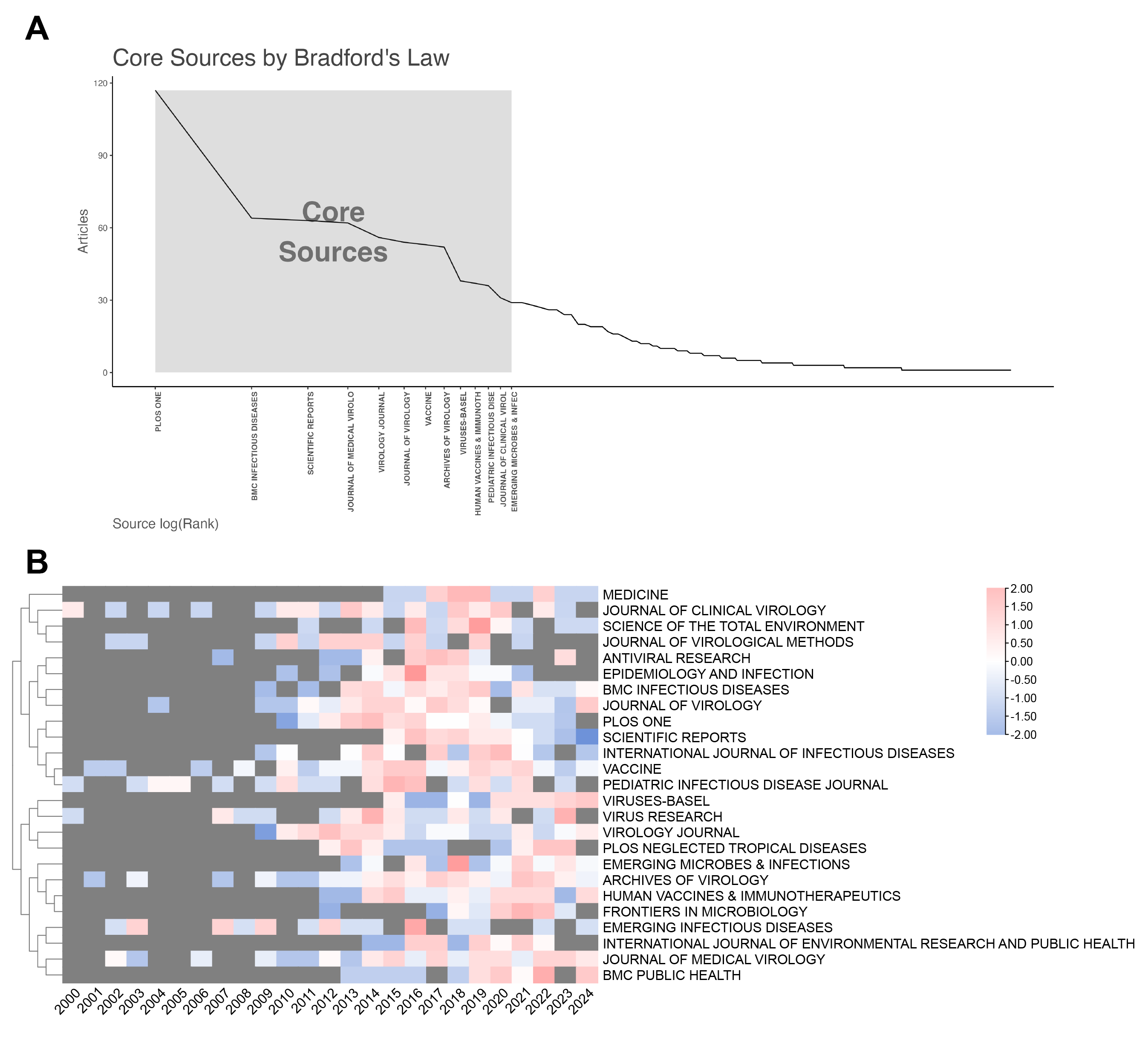


**Supplement Figure 2**

**(A)** Bradford’s Law can be applied to academic journals in the field, as evidenced by the grey-shaded area in the figure. This area encompasses the journals that have made significant contributions to the field, arranged in descending order based on the number of articles. The visualization overlays the publication volume of journals, with color intensity indicating the volume of publications. **(B)** Heatmap illustrating the correlation between journals, grouping journals with high content similarity in similar time periods and showing their publication trends on the timeline.

**
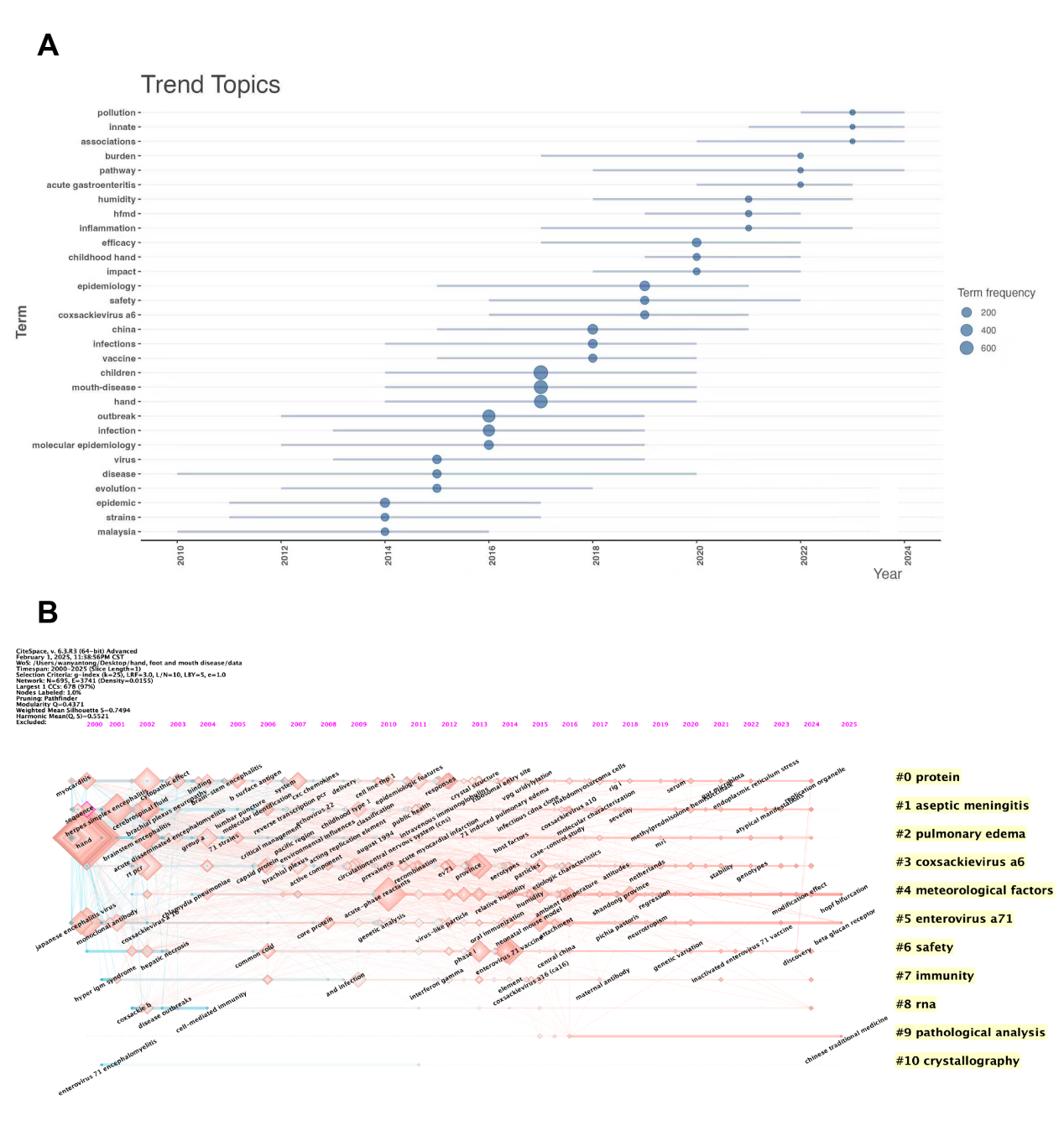
**

**Supplement Figure 3**

**(A)** The visual analysis depicts the duration of popularity of the top thirty keywords ranked by frequency of occurrence from 2010 to 2024. In this visualization, the length of the line indicates the duration of popularity, while the size of the dots indicates the frequency of occurrence in chronological order. (**B)** The keyword timeline presents clusters horizontally, with the size of each cluster inversely proportional to its number, where #0 denotes the largest cluster. Node size indicates the frequency of co-citation, and connections between nodes signify co-citation relationships. The year of node occurrence signifies when they were initially co-cited.


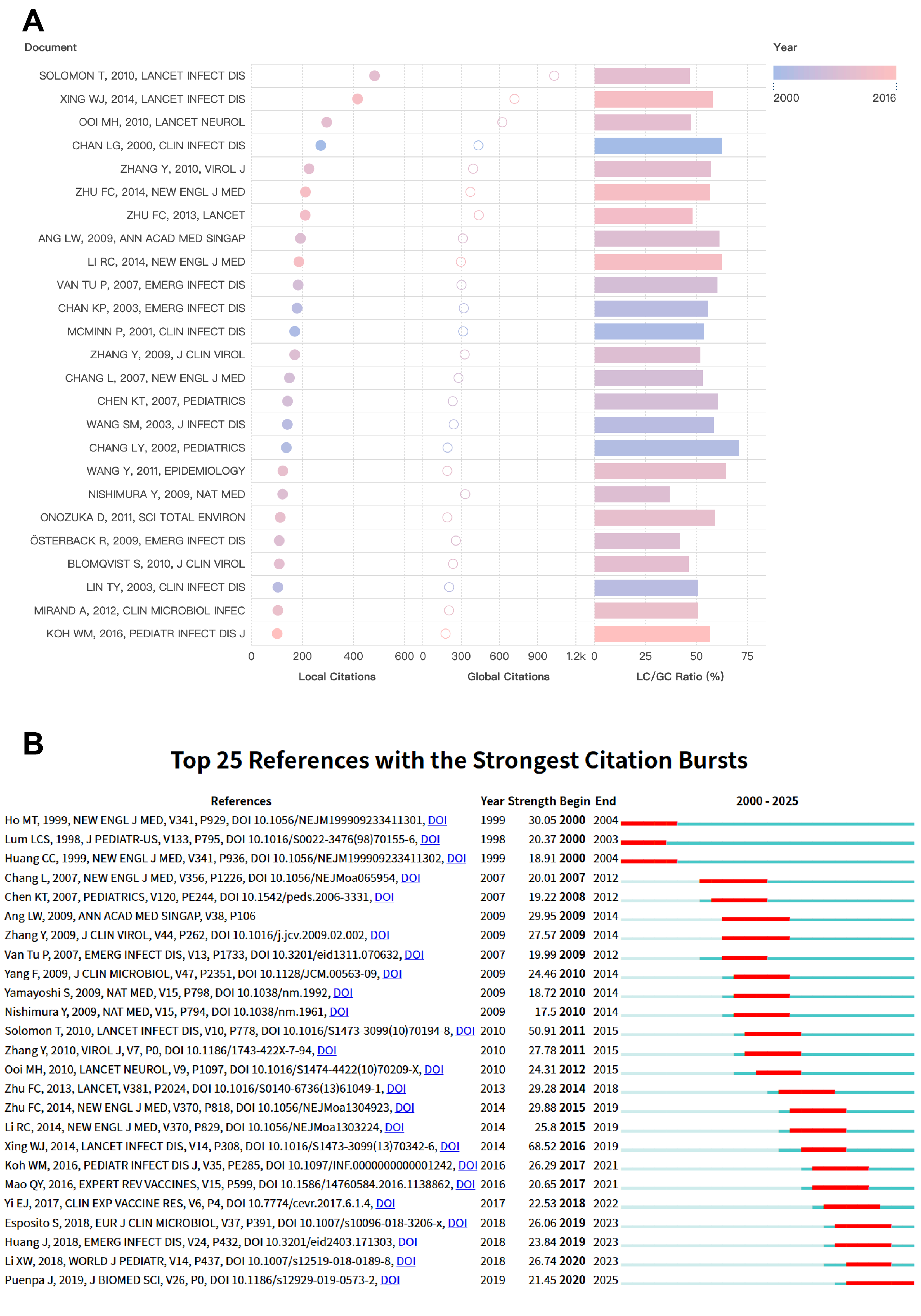


**Supplement Figure 4**

**(A)** The citations of the top 25 most cited documents, visualizing the percentage of citations in the field to the total citations, reflecting the impact of the article. **(B)** The diagram illustrates the 25 primary references characterized by pronounced bursts of citations, denoted by red spikes on the timeline. These spikes signify sudden surges in citation counts, signaling pivotal moments of emerging crucial questions or solutions within the field.
